# Biapenem Inactivation by B2 Metallo β-Lactamases: Energy Landscape of the Hydrolysis Reaction

**DOI:** 10.1371/journal.pone.0055136

**Published:** 2013-01-24

**Authors:** Sharon H. Ackerman, Domenico L. Gatti

**Affiliations:** 1 Department of Biochemistry and Molecular Biology, Wayne State University School of Medicine, Detroit, Michigan, United States of America; 2 Cardiovascular Research Institute, Wayne State University School of Medicine, Detroit, Michigan, United States of America; Wake Forest University, United States of America

## Abstract

**Background:**

A general mechanism has been proposed for metallo β-lactamases (MβLs), in which deprotonation of a water molecule near the Zn ion(s) results in the formation of a hydroxide ion that attacks the carbonyl oxygen of the β-lactam ring. However, because of the absence of X-ray structures that show the exact position of the antibiotic in the reactant state (RS) it has been difficult to obtain a definitive validation of this mechanism.

**Methodology/Principal Findings:**

We have employed a strategy to identify the RS, which does not rely on substrate docking and/or molecular dynamics. Starting from the X-ray structure of the enzyme:product complex (the product state, PS), a QM/MM scan was used to drive the reaction uphill from product back to reactant. Since in this process also the enzyme changes from PS to RS, we actually generate the enzyme:substrate complex from product and avoid the uncertainties associated with models of the reactant state. We used this strategy to study the reaction of biapenem hydrolysis by B2 MβL CphA. QM/MM simulations were carried out under 14 different ionization states of the active site, in order to generate potential energy surfaces (PESs) corresponding to a variety of possible reaction paths.

**Conclusions/Significance:**

The calculations support a model for biapenem hydrolysis by CphA, in which the nucleophile that attacks the β-lactam ring is not the water molecule located in proximity of the active site Zn, but a second water molecule, hydrogen bonded to the first one, which is used up in the reaction, and thus is not visible in the X-ray structure of the enzyme:product complex.

## Introduction

Class B β-lactamases are metallo-enzymes requiring one or two Zn^2+^ ions for activity [1]. The first of these enzymes, BcII from *Bacillus cereus*, was identified in 1966, and for the following two decades remained the only known example and a biochemical curiosity. After 1980, metallo β-lactamases (MβLs) were found among an increasing number of clinical isolates, and started to disseminate on mobile genetic elements to major Gram negative pathogens [2]; MβLs are now a worldwide threat, because they hydrolyze all β-lactams with the exception of monobactams, and are not inhibited by serine β-lactamase inhibitors, such as clavulanic acid, tazobactam, and sulbactam. MβLs are particularly efficient as carbapenemases, which is very alarming because carbapenems are the antibiotics with the largest spectrum of activity, and are stable to hydrolysis by most of the serine β-lactamases [2]–[4]. MβLs are evolving rapidly and becoming progressively more effective and specialized against different antibiotics [5]–[7]; unfortunately, no clinically relevant inhibitors of these enzymes are yet available [2], [8].

**Figure 1:**
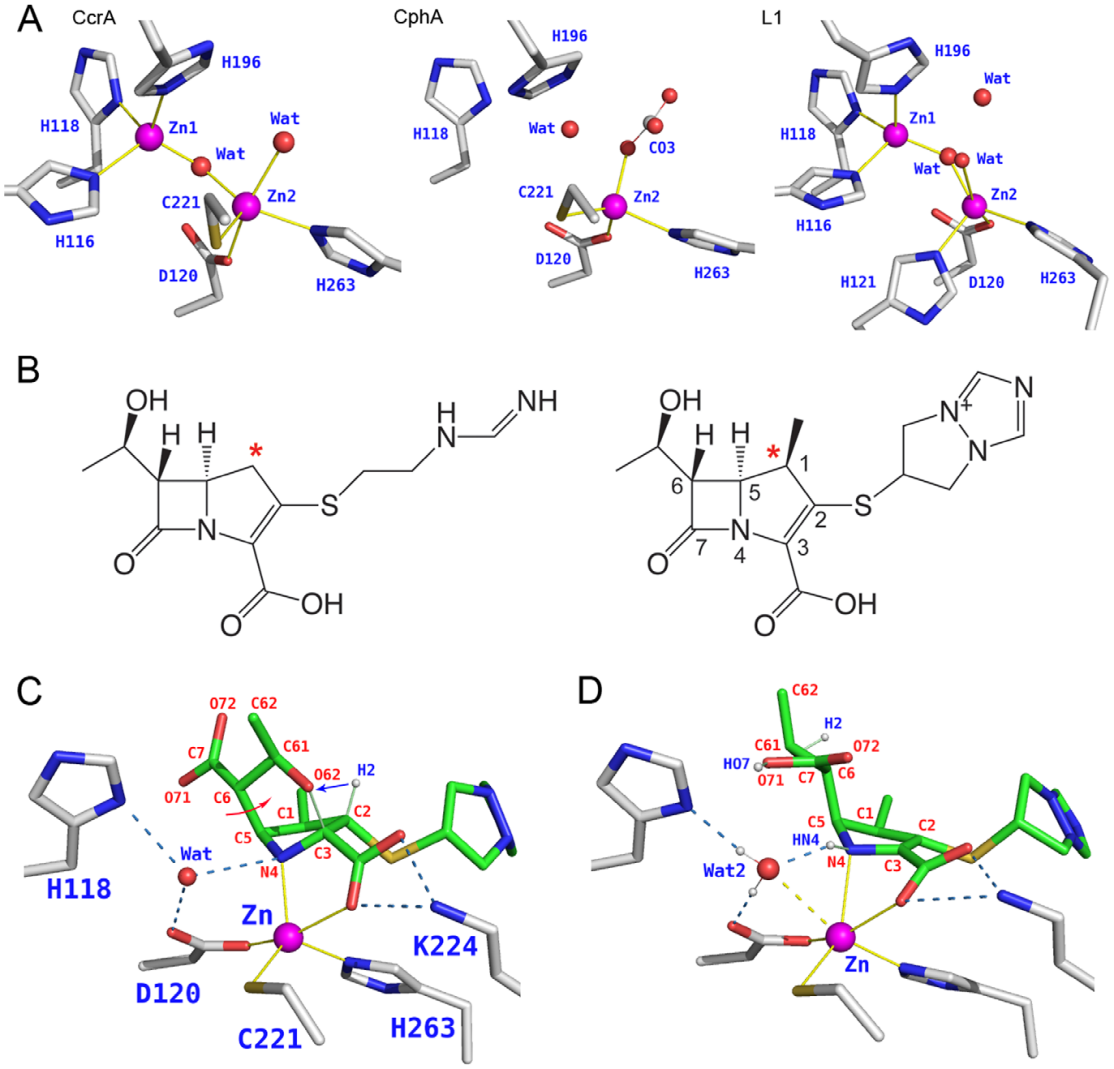
MβLs and some of their substrates. A. Zinc binding sites of B1 (CcrA), B2 (CphA with carbonate), B3 (L1) β-lactamases. Structural models are from PDB entries 1A7T, 1X8I, 2AIO. **B.** Two typical carbapenems: Imipenem (left), Biapenem (right). An asterisk marks the carbon atom that replaces the sulfur of penicillins. **C.** Active site of CphA in complex with a bicyclic form of hydrolyzed biapenem (PDB entry 1X8I). Zn^2+^ coordination and hydrogen bonds are shown as thin yellow lines and dashed blue lines. The two bonds formed during the rearrangement are shown as thin green lines. Biapenem numbering is in red with the exception of the hydrogen transferred from O62 to C2 during the rearrangement. Red and blue arrows indicate the dynamic constraints applied during QM/MM geometry optimization to reverse the rearrangement and generate the open-ring form shown in panel D. **D.** QM/MM optimized model of the CphA active site in complex with hydrolyzed biapenem: in this state both N4 and the C6 carboxylate are protonated (atoms HN4 and HO7). A water molecule hydrogen bonded to Asp120 and loosely coordinated to Zn^2+^ (2.9 Å, dashed yellow bond) is labeled Wat2 because it is near the Zn2 site (Panel A) and to distinguish it from a second water molecule (Wat1) that might be involved in the reaction. Zn^2+^ has only five strong ligands, in agreement with spectroscopic data [22].

Based on their sequence heterogeneity MβLs have been grouped into three subclasses (B1 to B3) [1]. Comparison of the tertiary structure of enzymes from the three subclasses reveals a common αβ/βα sandwich fold, and structure based sequence alignment has produced a standard numbering that allows easy comparison of the various sequences [9], [10]. All MβLs possess two potential Zn^2+^ sites (**Figure 1A**, **Table 1**), but differ in Zn^2+^ occupancies and coordination environments. In B1 enzymes one Zn is coordinated by His116, His118, His196 (Site 1 or Zn1), and the other Zn by Asp120, Cys221, His263 (Site 2 or Zn2). A water molecule or OH^−^ ion bridges the two Zn^2+^ ions. In B2 enzymes Site 1 is incomplete (having only His118 and His196) and without metal, and only Site 2 is occupied. In B3 enzymes Site 1 is unchanged, while at Site 2 His121 replaces Cys221 as a Zn ligand. B1 and B3 enzymes have maximum activity as di-zinc species [11]. In contrast, B2 enzymes exhibit maximal activity when bound to only one Zn, the binding of a second Zn ion being inhibitory [12], [13].

**Table 1:** Zn sites in MβLs.

Enzyme	Zn1 Site	Zn2 Site
B1	His116 His118 His196	Asp120 Cys221 His263
B2	His118 His196 (no Zn^2+^)	Asp120 Cys221 His263
B3	His116 His118 His196	Asp120 His121 His263

One significant obstacle to the design of effective inhibitors of MβLs has been the absence of X-ray structures that show the exact position of the antibiotic in the reactant state (the enzyme:substrate complex). Only recently, X-ray structures of one B2 enzyme (CphA from *Aeromonas hydrophila*) and one B3 enzyme (L1 from *Stenotrophomonas maltophilia*) in complex with product (the hydrolyzed β-lactam) have become available [14], [15]. A merger of information from biochemical (site-directed mutations, kinetics, spectroscopy [16]–[26]), structural (X-ray structures of the substrate-free enzymes [27], [28]), and computational studies (direct modeling of the substrate on the product, ligand docking, molecular dynamics (MD) often combined with quantum mechanical/molecular mechanical (QM/MM) simulations [29]–[40]) has been employed to predict the ionization state of key active site groups and the correct position of the antibiotic in the enzyme:substrate complex. On the basis of these studies a general mechanism has been proposed for MβLs, in which deprotonation of a water molecule at the Zn2 site results in the formation of a hydroxide ion that attacks the carbonyl oxygen of the β-lactam ring [17], [18], [24], [41], [42]. The original Zn-bound water/hydroxide ion is lost in this reaction and is replaced by a new solvent molecule that enters the active site before the next turnover.

An alternative mechanism that does not implicate the metal-bound water as the nucleophile that attacks the β-lactam ring has been proposed [36], but has not received general acceptance because the reactant state (RS) configuration was obtained by docking of biapenem in the structure of the ligand free enzyme followed by a refinement of the binding pose by MD [32], rather than from an experimental X-ray structure. Another example of the risks involved in using only docking and/or molecular dynamics to identify the correct position of the substrate in the active site is provided by a most recent study of the MβL AIM-1 from *Pseudomononas aeruginosa* [39] in which cefoxitin docking into the experimental X-ray structure led to the conclusion that Gln157 is essential for drug binding. However, subsequent mutation of Gln157 to Asn or Ala produced negligible effects on both *K*_m_ and *k*_cat_.

We have adopted a different strategy to identify the RS of the MβL reaction, which does not rely on substrate docking and/or molecular dynamics. Starting with the X-ray structure of the enzyme:product complex, the protonation states of the product and of key groups in the active site are assigned in such a way that these molecules contain all the atoms expected to be present in the enzyme:substrate complex at the RS. Then, a QM/MM scan is used to drive the reaction uphill from product back to reactant. As the reaction is driven backward also the conformation of the enzyme reverts to that of the reactant state. In so doing we actually generate the enzyme:substrate complex from product and avoid the uncertainties associated with modeling the RS.

## Results

### Driving the Reaction Backward

We studied the reaction of biapenem hydrolysis by CphA from *Aeromonas hydrophila*, a B2 type MβL. CphA is a strict carbapenemase with negligible activity against penicillins and cephalosporins. Carbapenems are structurally very similar to penicillins, but with the sulfur atom in position 1 of the structure replaced by a carbon atom (**Figure 1B**).

The structure of N220G CphA (∼½ the activity of wild type) was determined by Garau *et al.* ([14], PDB entry 1X8I) in complex with a form of hydrolyzed biapenem that has undergone a molecular rearrangement [14], [22] such that oxygen atom O62 forms a 6-membered ring (N4-C5-C6-C61-O62-C3) that replaces the original β-lactam ring (**Figure 1C**). While this bicyclic compound is the product of a minor pathway in the enzyme active site [43], [44], in a prequel to this study [44] we have shown that it can be formed at a higher rate in solution from a spontaneous cyclization of hydrolyzed biapenem, after which it binds back to the enzyme and acts as a product inhibitor [22].

Since the bicyclic compound shown in **Figure 1C** is a rearranged form of the product of β-lactam ring hydrolysis, starting from the structure in PDB entry 1X8I, in our previous work on CphA using a combination of QM/MM and metadynamics simulations we generated a form of the enzyme:product complex in which the steps of the post-hydrolysis reactions have been reversed [44]. For example, by transferring a proton from C2 to O62, adding protons to N4 and O71, and by rotating the carboxyethyl group around the C5–C6 bond, we effectively recreated the form of hydrolyzed biapenem that precedes the rearrangement, and provided a more realistic view of CphA in the enzyme:product state (**Figure 1D**) [44]. This modified structure served as the starting point for the application of a series of 2-dimensional QM/MM scans to generate the potential energy surfaces (PESs) corresponding to a large variety of possible reaction paths (**Table 2**). In these studies, the QM region was treated by density functional theory (DFT) [45] as described in [44] (see also Methods). Earlier computational studies of MβLs have more often represented the QM region at the semi-empirical level [30], [32], [35]–[38], [46], [47].

**Table 2:** Active site configurations at the PS in different QM/MM simulations.

QM/MMSimulation	N4^BIA^C6-COO^BIA^	H118	H196	D120	WAT2	H-bond acceptorfrom WAT2	H-bond donorto WAT2	Barrier height(kcal/mol)	Figure
**1**	NH4 COOH	HIE	HIE	O^–^	H_1_O^–^	D120(O)	BIA(HN4)	20	**S1**
**2**	N4^–^ COOH	HIE	HIE	O^–^	H_1_OH_2_	H118(ND1) BIA(O71) BIA(N4) D120(O)	H196(HE2)	15	**2A**
**3**	N4^–^ COOH	HIE	HIE	OH	H_1_OH_2_	H118(ND1) BIA(N4) BIA(O71)	D120(HD1) H196(HE2)	12	**2B**, **5**
**4**	NH4 COOH	HIE	HIP	O^–^	H_1_O^–^	D120(O)		24	**S2A**
**5**	NH4 COOH	HIP	HIP	O^–^	H_1_O^–^	H118(ND1) BIA(N4)	D120(HD1) H196(HE2)	30	**S2B**
**6**	NH4 COOH	HIE	HIE	O^–^	n.a.	n.a.	n.a.	30	**S2C**
**7**	NH4 COO^–^	HIE	HIE	O^–^	n.a.	n.a.	n.a.	∼60	**S2D**
**8**	NH4 COO^–^	HIE	HID	O^–^	n.a.	n.a.	n.a.	∼9^a^	**S3**
**9**	NH4 COOH	HIP	HIE	O^–^	n.a.	n.a.	n.a.	15	**3**
**10**	N4^–^ COOH	HIE	HID	O^–^	H_1_OH_2_	D120(O) BIA(O71) BIA(N4)		∼17	**S4**
**11**	N4^–^ COOH	HIE	HID	O^–^	H_1_O^–^	H118(ND1)	C6OH_BIA_	46	**S5A**
**12**	N4^–^ COOH	HIE	HIE	OH	H_1_OH_2_	H118(ND1) BIA(O71)	D120(HD1) H196(HE2)	23	**S5B**
**13**	NH4 COO^–^	HIE	HID	OH	H_1_OH_2_	H196(NE2) H118(ND1)	D120(HD1)	26	**S5C**
**14**	NH4 COOH	HIE	HID	OH	H_1_OH_2_	H196(NE2) H118(ND1)	D120(HD1) BIA(HN4)	∼13	**4**

Details of simulations 1, 4–8, 10–13 are provided in **Text S1** and **Figures** **S1**, **S2**, **S3**, **S4**, **S5**.

a The RS structure in this simulation does not correspond to a known antibiotic.

A principal aspect of the generally accepted mechanisms of MβL B2 enzymes (e.g. CphA) is that the active site Zn^2+^ favors the deprotonation of the nearby water molecule hydrogen bonded to Asp120: the resulting hydroxide ion would then attack the β-lactam carbonyl carbon (C7) generating a tetrahedral intermediate [14], [41]. Alternatively, for MβL B1 and B3 enzymes, the transition state is predicted to be a ring-opened, unprotonated species [18], [25], [41], [48]. Either way, the transfer of a proton to the ring nitrogen (N4) ultimately generates the product.

However, some considerations suggest a possible different source for the nucleophile in the reaction. First, a hydroxide ion generated by proton donation to either His118 or Asp120 would be too strongly coordinated to Zn^2+^ for it to assume the required position for attack on the β-lactam carbonyl. Second, if the atoms of the water molecule hydrogen bonded to Asp120 are ultimately incorporated in the product, why is a water molecule still visible near Asp120 (Wat in **Figure 1C**) in the X-ray structure of the enzyme:product complex? Since this water molecule is not accessible to bulk solvent when the product is bound, one possible explanation is that in the crystal of CphA, product-bound enzyme is in equilibrium with the product-free state, and that water becomes bound before the product re-binds in the active site. An alternative explanation is that the state shown in **Figure 1D** is the real product state, and that the water labeled Wat2 is not a new solvent molecule that occupies the position of the original nucleophile, but a *second* water molecule that also participates in the reaction. The real nucleophile would be some other water molecule (Wat1) that was present in the reactant state, but was used up in the reaction to form the carboxylate hydroxyl atoms (O71 and HO7 in **Figure 1D**) and the proton on N4 (HN4 in **Figure 1D**).

We have investigated this possible scenario by calculating the potential energy surface (PES) for a set of reactions in which starting from different ionization configurations of the active site (**Table 2**) in the product state (PS) we generate back the reactant state (RS). During this process the active site gradually converts from its geometry and ionization state at the PS to the geometry and ionization state at the RS. It is important to notice that although what initially changes at each scan point are only the lengths of the bonds (used here as collective variables) that break or form during the hydrolysis of biapenem, during the following geometry optimization the entire protein and solvent reorganize in response to the changes of the collective variables. As a consequence, the final PES includes also the contribution from the reorganization energy of the protein and solvent, and reflects the conformational changes of the protein during the course of the reaction. It is also important to keep in mind that once the PES is generated it can be analyzed regardless of the reaction direction. Thus, in our case, we will focus on the *forward* reaction corresponding to biapenem hydrolysis.

### Energy Landscape of the Hydrolysis Reaction under Different Active Site Configurations

The configurations of the active site at the PS that were analyzed by QM/MM simulations are summarized in **Table 2**. These include different ionization states of His118, Asp120, His196, of biapenem (at the N4 atom and the C6 carboxylate), and of the water molecule located at Site 2 (**Figure 1D**). Different tautomeric states (HIE, proton on Nε; HID, proton on Nδ) were also considered for His118 and His196. Among the various PESs calculated in this study, reaction barriers along the path from RS to PS consistent with the barrier determined experimentally (∼14 kcal/mol [14]) were observed on the surfaces derived from simulation 2,3,9, and 14 (**Table 2**). Details of these simulations are provided in the following sections, while details of the other simulations (corresponding to higher reaction barriers) are provided as (**Text S1, Figures S1, S2, S4, S5**). In one case (**Simulation 8**) a barrier of only 9 kcal/mol was calculated, but the RS in this simulation corresponds to a compound that is different from biapenem (and is not a known antibiotic), and thus it does not reflect a reaction that would normally be catalyzed by CphA; details of this simulation are also provided as (**Text S1, Figure S3**).

#### Active site configuration 2

**Figure 2:**
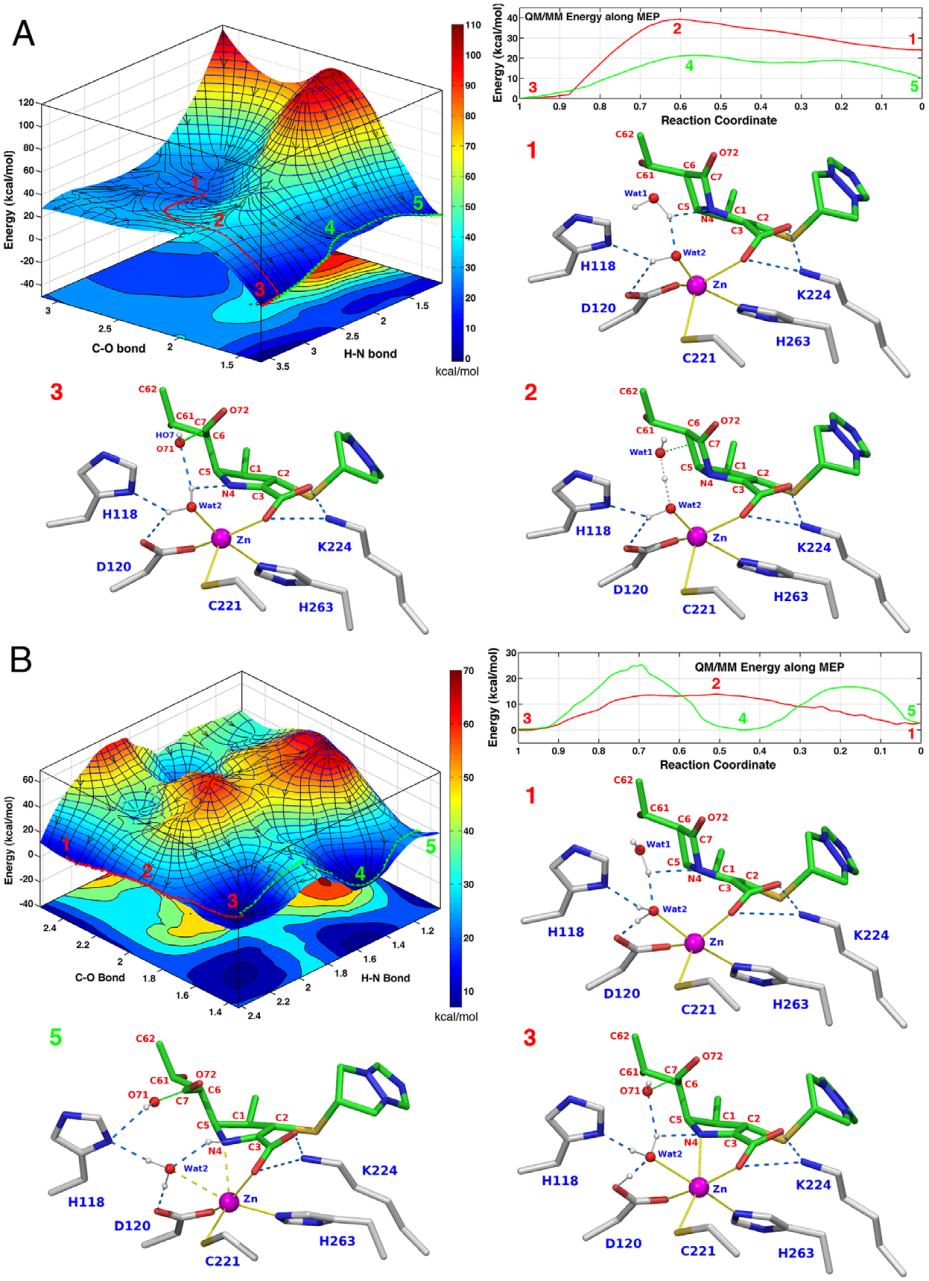
PES and active site configurations for the reaction corresponding to Simulations 2–3 in Table 2. **A.** ***Top left*** **.** PES of the reaction calculated using the C–O and H–N bonds as scanning coordinates. Two possible product states are visible on the PES corresponding respectively to hydrolyzed biapenem with unprotonated (labeled 3) or protonated N4 (labeled 5). The minimum energy path (MEP) from RS to unprotonated PS is traced by a red string. The MEP from this PS to the PS in which N4 is protonated is shown as a green string. ***Top right*** **.** QM/MM energy values along the two MEPs. RS and ionized PS are separated by a barrier of ∼15 kcal/mol, and the reaction is strongly exergonic (−24 kcal/mol). Protonated product is ∼10 kcal/mol higher in energy than unprotonated product and a barrier of ∼20 kcal/mol separates the two states. The other three insets show the configurations of the active site corresponding to the RS, TS, and PS along the red MEP on the PES. At the RS (inset 1) Wat2 is present as a hydroxide ion. Coincident with the formation of a tetrahedral TS, a proton is shared between Wat1 and the hydroxide ion (inset 2). Concurrent with the opening of the ring (inset 3) the proton is transferred to Wat2. Thus, the reaction proceeds through the formation of a tetrahedral TS, and the rate-limiting step is the concurrent formation of the C–O bond while the C–N bond is broken. There is no proton transfer to His118, His196 or Asp120. **B.** ***Top left*** **.** PES of the reaction calculated using the C–O and H–N bonds as scanning coordinates. Two possible product states are visible on the PES corresponding respectively to hydrolyzed biapenem with unprotonated or protonated N4. The MEP from RS to unprotonated PS is traced by a red string. The MEP from this PS to the PS in which N4 is protonated is shown as a green string. ***Top right*** **.** QM/MM energy values along the two MEPs. RS (labeled 1) and ionized PS (labeled 3) are separated by a barrier of ∼12 kcal/mol, and the reaction is only slightly exergonic (−3 kcal/mol). Protonated product (labeled 5) is isoenergetic with unprotonated product, but a barrier of ∼25 kcal/mol separates the two states. The other three insets show the configurations of the active site corresponding to the RS, ionized PS, and protonated PS. At the RS (inset labeled 1) both Wat1 and Wat2 are fully protonated. At the ionized PS a proton has been transferred from Wat1 to Wat2, and from Wat2 to Asp120 (inset labeled 3): thus Wat2 remains fully protonated. At the protonated PS (inset labeled 5) a proton has been transferred from Wat2 to biapenem N4, and from Asp120 to Wat2: thus also in this case Wat2 remains fully protonated. The most favorable reaction proceeds through the formation of a tetrahedral TS (labeled 2 on the PES), and the rate-limiting step is the concurrent formation of the C–O bond and the breaking of the C–N bond.

In this simulation, at the PS with the lowest energy biapenem N4 is deprotonated, Wat2 is not ionized, Asp120 is deprotonated, and His118, and His196 are neutral (**Table 2** **, Simulation 2**). The C_BIA_–O_BIA/WAT1_, HN_BIA_–N_BIA_ distances were used as the scanning coordinates in the construction of the PES (the latter is the H–N bond that is formed when the amide nitrogen of the product is protonated). In this case, a barrier of ∼15 kcal/mol (at C–O≅2.0 Å; labeled 2 in **Figure 2A**) separates the reactant (C–O≅2.5 Å; labeled 1) from the product: this barrier corresponds to an attack by WAT1 on the carbonyl carbon (C7) of the lactam ring. Past the TS, the reaction leads to either a protonated (C–O≅1.4 Å and H–N≅1.03 Å; labeled 5) or unprotonated (C–O≅1.4 Å and H–N≅3.6 Å; labeled 3) product. However, the energy level of the unprotonated product is ∼10 kcal/mol lower than that of protonated product and a barrier of ∼20 kcal/mol (labeled 4) separates the two states (**Figure 2A**).

#### Active site configuration 3

A very similar configuration of the active site was explored in **Simulation 3** (**Table 2**), the only difference with respect to Simulation 2 being that Asp120 was protonated in the product state with the lowest energy. In this case a barrier of 12 kcal/mol separates the RS (C–O≅2.4 Å and H–N≅2.4 Å; labeled 1 in **Figure 2B**) from the unprotonated product (C–O≅1.5 Å and H–N≅2.2 Å; labeled 3). Formation of a protonated product (C–O≅1.5 Å and H–N≅1.05 Å; labeled 5) occurs over a much higher barrier of ∼25 kcal/mol.

#### Active site configuration 9

**Figure 3:**
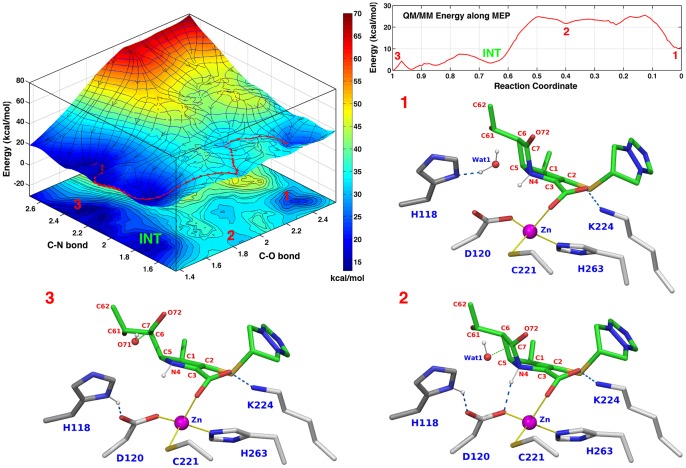
PES and active site configurations for the reaction corresponding to simulation 9 in Table 2. **Top left.** PES of the reaction calculated using the C–O and C–N bonds as scanning coordinates. The MEP from RS to PS is traced by a red string. **Top right.** QM/MM energy values along the MEP: the reaction is exergonic (−10 kcal/mol), and an extended plateau (at ∼15 kcal/mol, labeled 2) consisting of 2–3 poorly differentiated TSs (or intermediates) separates RS (labeled 1) from PS (labeled 3). The other three insets show the configurations of the active site corresponding to the RS, TS, and PS along the MEP. At the RS (inset 1) Wat2 is absent, and Wat1 is hydrogen bonded to His118. Coincident with the formation of a tetrahedral TS, a proton is transferred from Wat1 to His118 (inset 2). Thus, the rate-limiting step is the formation of a tetrahedral intermediate (labeled INT on the PES) with a stretched C–N bond (1.8 Å); a barrier of only ∼5 kcal/mol separates this intermediate from the fully hydrolyzed product. The Zn ion retains a tetrahedral coordination throughout the reaction.

The reaction was also simulated under conditions consistent with the generally accepted mechanism of B2 MβLs, which predicts that only one water molecule (initially positioned near Asp120 at the Zn2 site) is the nucleophile that attacks the β-lactam ring. Since this water molecule is consumed in the reaction to form the product, testing this standard mechanism requires that the Zn2 site be left empty at the PS when calculating the PES in a reverse scan from PS to RS. Four different ionization states of the PS were simulated (**Table 2**, **Simulations 6–9**) to test this condition. As previously mentioned, in **Simulation 8** the RS is not biapenem (**Text S1**, **Figure S3**), and thus this simulation does not reflect a natural function of CphA. Only in one of the other simulations the calculated reaction barrier was of the same magnitude as that found experimentally (∼14 kcal/mol). In this case, biapenem N4 and C6 carboxylate, and His118 were all protonated at the PS (**Table 2**, **Simulation 9**). Analysis of the PES (**Figure 3**) reveals that a tetrahedral intermediate (C–O≅1.45 Å, C–N≅1.76 Å, H–N≅1.03 Å, labeled INT) is formed, and an overall barrier of ∼15 kcal/mol (large plateau around C–O≅1.85 Å, C–N≅1.6 Å, labeled 2) separates RS (C–O≅2.4 Å, C–N≅1.66 Å, H–N≅1.03 Å; labeled 1) from PS (C–O≅1.5 Å, C–N≅2.4 Å, H–N≅1.03 Å; labeled 3) This is the simulation that most closely reflects the standard mechanism (as there is no water in the active site at the PS): however, at the RS, it is a water molecule near the Zn1 site (Wat1), not the Zn2 site that generates the nucleophile in the reaction.

#### Active site configuration 14

**Figure 4:**
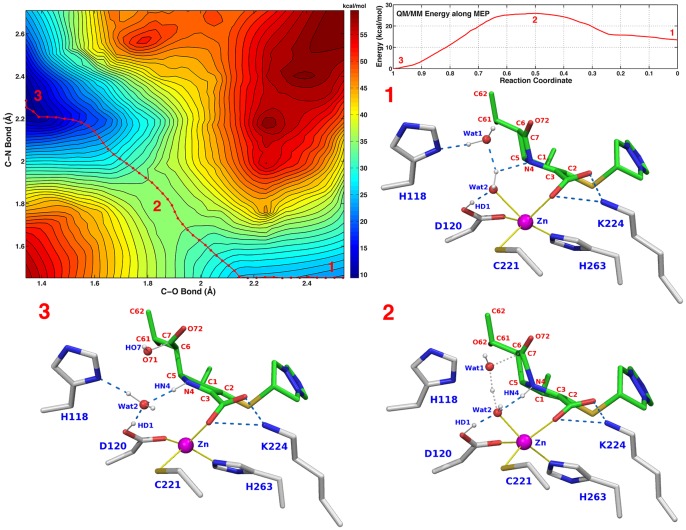
Active site configurations for the reaction corresponding to simulation 14 in Table 2. **Top left.** PES of the calculated using the C–O and C–N bonds as scanning coordinates. **Top right.** QM/MM energy values along the MEP: the reaction is exergonic (−14 kcal/mol), and a barrier of ∼13 kcal/mol (labeled 2) separates RS (labeled 1) from PS (labeled 3). Panels labeled 1–3 show the configurations of the active site at the RS, TS, and PS labeled 1,2,3 on the PES.

Finally, we have studied additional PS configurations in which Asp120 was protonated (as suggested in [49]) and donated a hydrogen bond to Wat2. Although 3 different configurations were examined (**Table 2**, **Simulation 12–14**), only in one case the reaction barrier was of magnitude comparable to the experimental one. In this configuration (**Table 2**, **Simulation 14**) biapenem C6 carboxylate (which originates from water attack on the carbonyl carbon of the β-lactam ring) was protonated at the PS, and Wat2 donated hydrogen bonds to His118 and His196. The calculated barrier (at C–O≅1.85 Å, C–N≅1.83 Å) for the reaction from RS (C–O≅2.5 Å, C–N≅1.45 Å) to PS (C–O≅1.34 Å, C–N≅2.3 Å) is ∼13 kcal/mol (**Figure 4**). Configurations of the active site at the RS, TS, and PS for this simulation are shown in panels 1–3 of **Figure 4**.

### Free-energy Profile of the Hydrolysis Reaction in the Active Site Configuration 3

**Figure 5:**
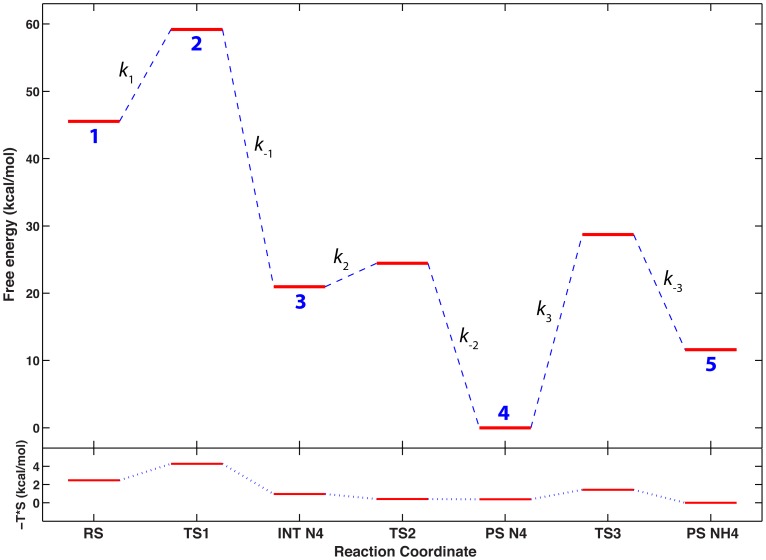
Free energy profile for the reaction corresponding to Simulation 3 in **Table 2**. Upper quadrant. Stationary and TS points are represented as red thick horizontal lines connected by dashed blue lines; numbers in blue (1–5) below each red line correspond to the same numbers on the PES of **Figure 2B**. Rate constants for the forward and reverse reaction at each step are defined next to each transition: values of these rate constants are reported in **Table 3**. The reaction coordinate axis is in arbitrary units and the stationary points are marked as follows: RS is biapenem; INT N4 is an intermediate conformation of the active site in which the β-lactam ring of biapenem is already open and N4 is ionized; PS N4 is a slightly changed conformation of the active site in which Wat2 becomes closer to hydrolyzed biapenem, but N4 is still ionized; PS NH4 is the open-ring form of biapenem with N4 protonated. **Lower quadrant.** Changes in the entropic contribution (-T*S) to the free energy profile shown in the upper quadrant. Both the free energy and the entropy profile are not on an absolute scale, but were shifted such that their smallest value would correspond to 0 on the energy axis.

Although the PESs shown in the previous section are based only on QM/MM energies, zero point energy and entropic contributions are expected to be very similar for all the active site configurations analyzed. Furthermore, in our previous QM/MM work on CphA [44] we have shown that entropic contributions to the reactions that occur in the active site of this enzyme are very small (at most 2–3 kcal/mol). Therefore, identification of the most likely mechanisms on the basis of the QM/MM PESs is expected to be substantially accurate. However, calculation of a reliable kinetic model of the reaction of inactivation of biapenem by CphA requires the calculation of free energies from which rate constants for all reversible steps can be derived. For this reason, a complete vibrational analysis of the transition (TS) and stationary points (SP) on the PSE derived from **simulation 3** (which has the lowest overall energy barrier for the hydrolysis step) was carried out to convert the values of electronic energy into free-energy values. The association of each TS with the two neighbor SPs was verified by an Intrinsic Reaction Coordinate (IRC) analysis in the forward and reverse direction using the method of Gonzales and Schlegel [50]–[52]. The free energy profile of the reaction so derived (**Figure 5** **)** is qualitatively similar to the electronic energy profile of **Figure 2B** **,** and shows again the largest barrier to be the step of N4 protonation. Rate constants corresponding to the three steps of the reaction from biapenem to its hydrolyzed form with N4 protonated (**Figure 5** **),** are reported in **Table 3**.

**Table 3:** Free energy differences and rate constants for individual steps in the hydrolysis reaction of biapenem corresponding to Simulation 3 in **Table 2**, as derived from the free energy profile in **Figure 5**.

Reaction	ΔG^‡^ (kcal/mol)	ΔG^0^ (kcal/mol)	Rate Constant (s^−1^)	Rate Constant Definition
RS→INT N4	13.66	−24.57	599.2	*k*_1_
INT N4→ RS	38.23		5.70×10^−16^	*k*_−1_
INT N4→ PS N4	3.50	−20.96	1.67×10^10^	*k*_2_
PS N4→ INT N4	24.46		7.12×10^−6^	*k*_−2_
PS N4→ PS NH4	28.73	11.61	5.25×10^−9^	*k*_3_
PS NH4→ PS N4	17.12		1.73	*k*_−3_
RS→PS NH4		−33.91		

## Discussion

A general mechanism for MβLs has been proposed, in which one or both Zn ions act as Lewis acids decreasing the p*K* of a nearby water molecule so that the resulting hydroxide ion attacks the carbonyl oxygen of the β-lactam ring. In some cases, the rate-limiting step is believed to be the cleavage of a tetrahedral intermediate concurrent with protonation of the nitrogen of the lactam ring [24]. In other cases, it was proposed that cleavage of the C−N bond leads to a second intermediate, in which the leaving amide moiety in deprotonated form is coordinated to the Zn2 ion. Protonation of this negatively charged intermediate would then be the rate-limiting step in the catalytic cycle [17], [18], [41], [42], [53]. In all cases, the original Zn-bound water/hydroxide ion is used in the reaction and is replaced by a new solvent molecule entering the active site at the beginning of the next cycle.

Regardless of the specific mechanism proposed for different enzymes, since there are no X-ray structures of MβLs that show the exact position of the antibiotic in the reactant state (the Michaelis complex), all the earlier computational studies were based either on the structures of the substrate-free enzymes, in which the substrate was docked computationally, or on the structures of the enzyme:product complex, in which the product was simply replaced with the substrate, and its binding pose was improved by molecular dynamics. In this study we have adopted a strategy to identify the reactant state of the reaction, which does not rely on docking or MD. We start with the experimental structure of the enzyme in complex with product. The protonation states of the product and of key groups in the active site are assigned in such a way that these molecules contain all the atoms that are expected to be present in the enzyme substrate complex at the reactant state. Then, a QM/MM coordinate scan is used to drive the entire ensemble (protein+solvent+ligands) uphill from PS back to RS. In essence, instead of modeling the enzyme:substrate complex, we generate it from the enzyme:product complex.

Throughout this study this basic idea was specifically applied to the analysis of the hydrolysis of biapenem by the B2 metallo β-lactamase CphA. However, on a more global scale, we submit that this computational strategy is of general value and applicability in a large number of mechanistic studies. For example, it is usually difficult to obtain crystal structures of enzymes in complex with their natural substrate(s), because enzymes in the crystalline state typically retain significant levels of activity. Without resorting to time-resolved crystallography (which requires crystals of rarely achievable quality and is mostly limited to compounds that can be released by photoactivation), incubating crystals with substrate(s) more often yields the structure of the product. Thus, the procedures used in this study can be employed to obtain accurate simulations of enzymatic reactions in many other cases in which the product state is known, but information on the reactant state is lacking, and less rigorous forms of modeling would otherwise be used to obtain this information.

Among the various PESs calculated in this study, the lowest energy reaction paths from RS to PS were observed on the surfaces derived from simulation 2, 3, 9, and 14 (**Table 2**). In these cases the energy barriers were either consistent with (**Simulations 2, 9**) or even lower (**Simulations 3, 14**) than the experimentally determined barrier (∼14 kcal/mol [14]). A common feature of the reactions described by these PESs is that Wat1, not Wat2, is the nucleophile that attacks the carbonyl oxygen of the β-lactam ring. Although not directly involved in the reaction, Wat2 plays also an important role in it, by participating in proton relays that lead to deprotonation of Wat1, and attack by the resulting hydroxide ion on the lactam ring. It is worth noting that a water molecule in a very similar position to that of Wat1 at the RS in the QM/MM simulations is also present in the metallo β-lactamase Sfh from *Serratia fonticola*, the only other B2 MβL for which an X-ray structure (PDB entry 3Q6VA) is available. The position of Wat1 is also close to the Zn1 site, which in CphA is not occupied by a metal ion (**Table 1**, **Figure 1**). This particular location of Wat1 may explain the observation that the binding of a second Zn at the Zn1 site (by perhaps disturbing the exact positioning of Wat1) suppresses the activity of B2 MβLs [12], [13]. A model for biapenem hydrolysis by CphA, in which a water molecule different from that hydrogen bonded to Asp120 is the nucleophile in the reaction, was proposed also by Simona *et al.* [32], [36] on the basis of substrate docking, MD, and QM/MM simulations. However in Simona’s model this second water molecule is also coordinated to Zn^2+^ at the Zn2 site, and biapenem interacts only indirectly with the metal via this water.

Of the four PESs with energy barriers comparable to the barrier determined experimentally (∼14 kcal/mol), only one (**Simulation 9** in **Table 2**, **Figure 3**) was calculated starting from a PS configuration in which Wat2 is absent: thus this PES conceptually reflects the mechanism of MβLs that has gained popularity, according to which only one water molecule is involved in the reaction. However, also in this case, the catalytic water occupies the Zn1 site, and not the Zn2 site.

While the PESs with the lowest barriers are all potentially good candidates for representing the ‘true’ reaction mechanism, they are not all equally likely, as only a few of them are consistent with the chemistry that occurs after the hydrolysis of the β-lactam ring. In the reaction of biapenem inactivation by CphA, after the β-lactam ring is opened, the carboxyl group generated by the hydrolytic process and the hydroxyethyl group rotate around the C5–C6 bond, assuming a new position that allows a proton transfer from the hydroxyethyl group to C2, and a nucleophilic attack on C3 by the oxygen atom of the same side-chain. This process leads to the formation of the bicyclic compound that was originally observed in the X-ray structure of CphA in complex with product [14], and also in the hydrolysis of imipenem by ImiS [22]. In previous work, in which we studied the landscape of these post-hydrolysis reactions, we have shown that the expected rotation of the hydroxyethyl group and the following cyclization step can occur at a rate comparable to that observed experimentally for the enzymatic inactivation of biapenem only if the hydrolysis reaction leaves the N4 nitrogen of the β-lactam ring unprotonated [44]. On this basis, the most likely mechanisms for biapenem hydrolysis by CphA are those corresponding to the PESs and active site configurations derived from **simulations 2**, **3** (**Figure 2**).

In this context, it is worth noting that the analysis of different reaction mechanisms presented in this study is primarily based on the calculation of electronic QM/MM PESs, which are only an approximation of the free energy PES. In particular, although all the points of the QM/MM PESs were derived through relaxed scans, there was no extensive sampling of the degrees of freedom perpendicular to the reaction coordinates. This problem was partially alleviated by the fact that the PESs were 2-dimensional and different scanning coordinates (e.g. C–O, C–N, H–N) were used, so that a large number of additional phase-space points outside the minimum energy paths (MEPs) on the PESs were visited. A more accurate estimate of the reaction free energy path was achieved for the ‘most likely’ reaction mechanism (**Configuration 3** in **Table 2**, **Figure 2B**) by deriving the enthalpic (H) and entropic contributions (-T*S) from the vibrational properties of individual stationary points and TSs on the PES (see Methods). Entropic contributions were small (less than 2.5 kcal/mol difference between RS and PS_NH4, **Figure 5** **)**, in agreement with several earlier studies of the entropic effects in enzymatic reactions [54]–[60], which found that most of the entropy changes occur during substrate binding, and that the remaining entropy changes as the reaction progresses toward the product(s) typically do not exceed 3 kcal/mol, and are not significantly different in the enzyme active site with respect to the same reaction in solution. However, even our best determination of free energies did not include sampling of the phase space for the regions of the ensemble outside the QM part, and was based only on end-point (SPs and TSs) calculations and not on a free-energy perturbation (FEP) [61] procedure, which would have been prohibitive at the level of theory (DFT-B3LYP) adopted in this study.

Notwithstanding these limitations, our study provides novel insights (like the role of Wat1) into the mechanism of B2 MβLs, and a reasonable estimate of the free energy profile of the hydrolysis of biapenem catalyzed by CphA. The common view that Wat2 is the nucleophile attacking biapenem may have affected negatively previous attempts to design inhibitors of B2 metallo β-lactamases. For example, it is possible that particular group substitutions in the β-lactam ring (e.g., the replacement of the ring carbonyl with a sulfone, like in β-sultams) would produce a displacement of Wat1 from the active site, thus completely blocking the reaction. Such displacement could also be advantageous from the standpoint of obtaining ligands with high affinity, because a significant contribution to the binding free energy comes from the release of localized water molecules in the binding pockets of biomolecules [62]. Thus, regardless of the role played by Wat1 in the hydrolysis reaction, the optimization of inhibitors for B2 MβLs may benefit from the introduction of chemical moieties that specifically attempt to displace this water from the active site. The fact that Wat1 occupies in the B2 types a position close to the second Zn ion in the other types of metallo β-lactamases, suggests that perhaps a common mechanism based on Wat1 is present in all MβLs, and that a common strategy to inhibit these enzymes may be possible.

**Figure 6:**
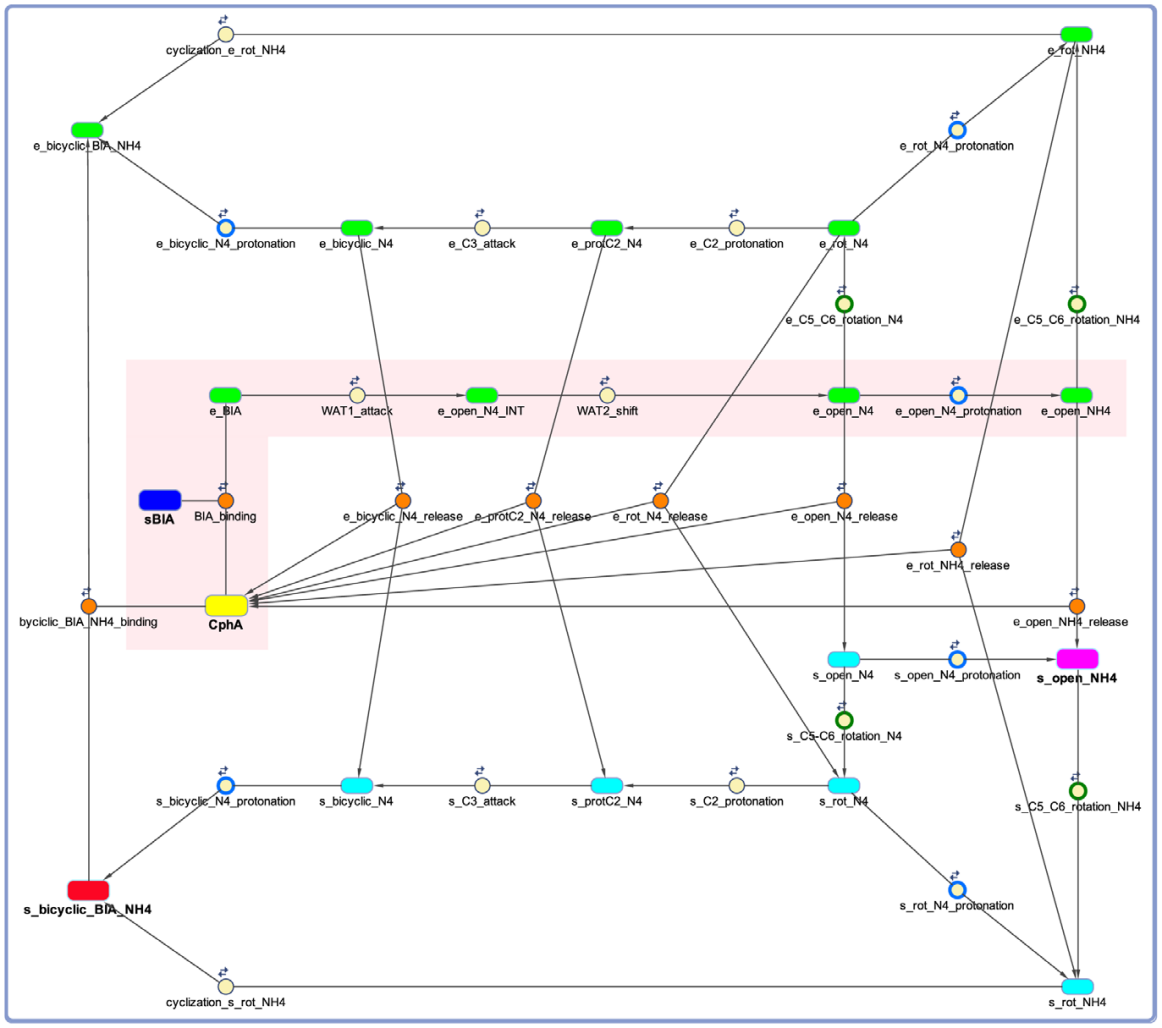
Kinetic model of biapenem inactivation by CphA. The model describes the binding of biapenem (blue box) to CphA (yellow box) and its subsequent conversion to an open-ring form (purple box) and to a bicyclic product (red box). Formation of the bicyclic derivative of biapenem occurs both in the enzyme (species labeled ‘e_’) and in solution (species labeled ‘s_’). All other enzyme-bound and bulk-solvent species are represented as rounded green and cyan boxes, respectively. Species with N4 ionized are labeled ‘_N4’, while those with N4 protonated are labeled ‘_NH4’. Reactions are shown as circles with arrows connecting the species involved. Orange-filled circles represent binding/release reactions; green-outlined circles represent rotations of the hydroxyethyl moiety around the C5–C6 bond; blue-outlined circles represent protonations of the N4 nitrogen. A pink shaded area highlights the reaction corresponding to **Simulation 3** in **Table 2** (see also **Figures 2B** and **5**). Parameters for all the other reactions were derived from [44]. Additional details about individual species, parameters, and reactions are provided as **Text_S2**.

In closing, we notice that the rate constants derived in this study for the steps of the hydrolysis reaction can be combined with those determined in our previous study [44] for the steps of the post-hydrolysis conversion of biapenem to a bicyclic product to generate a complete kinetic model of biapenem inactivation by CphA (**Figure 6**). A version of this model including all the kinetic laws is provided in Matlab SimBiology format (archive file **Model S1**) and SBML format (**Model S2**). The model, which includes two symmetric branches for the generation of the bicyclic derivative of Biapenem both inside the enzyme (green species in **Figure 6**) and in the solvent bulk-phase (cyan species in **Figure 6**), was used to simulate steady-state progress curves of biapenem inactivation by CphA (**Figure S6**): attesting to the overall accuracy of the rate constants derived from the QM/MM simulations, only small adjustments of the initially guessed *K*_d_ values for biapenem and its open-ring forms (values that are not available from experiments or from our calculations) were sufficient to obtain an almost perfect fit of the calculated initial velocities to the reported *k*_cat_ = 300 s^−1^ and *K*_m_ = 166 µM of CphA for biapenem [14] (**Figure S6**). This model can be conveniently used in future studies for further refinement of the kinetic parameters of the reaction of biapenem inactivation by CphA against both steady-state and pre-steady-state experimental observations.

## Methods

### QM/MM Simulations

QM/MM simulations [63] of the enzymatic hydrolysis of biapenem were carried out with Jaguar/Qsite (Jaguar, version 7.7, Schrodinger, LLC, New York, NY, 2010): an entire molecule of CphA from *Aeromonas hydrophila* in complex with the bicyclic form of hydrolyzed biapenem (as derived from the refined coordinates of the X-ray structure, PDB entry 1X8I) was solvated inside a cubic box of SPC [64] waters of 70 Å side, retaining all the original structural waters. For regions outside the active site, the most probable protonation state of histidines, and the optimal tautomeric states of arginines, glutamines, and histidines were determined using the Protein Preparation Wizard of the Schrodinger Suite, which optimizes the protein hydrogen bond network by means of a systematic, cluster-based approach. Results obtained with this protocol were consistent with those obtained by assuming pH = 7.0 and determining the protein p*K*_a_’s with PROPKA [65]–[67]. As for the ionization states of residues of the active site involved in the binding of the metal and the antibiotic we felt that studying only the ionization state predicted by computational methods was unsatisfactory, and that a more thorough investigation required testing various combinations of ionization states in different QM/MM simulations (see Results Section). After an initial geometry optimization (RMS deviation of the relaxed structure from the original crystal structure <0.3 Å), the added solvent and all hydrogen atoms in the protein and its ligands were equilibrated with 100 ps MD at 300 K in the NPT ensemble with periodic boundary conditions and SHAKE constraints [68] using the OPLS-AA force-field [69], [70]; during this short MD all heavy atoms in the protein and ligand were constrained to the positions attained during the previous geometry optimization. At this point the ensemble was readied for the QM/MM simulations under stochastic boundary conditions by removing any waters containing atoms farther than 26 Å from the N4 of biapenem (the center of the QM/MM system) or 6.0 Å from any other protein atom. Afterwards, residues or water molecules containing atoms farther than 24 Å from the N4 of biapenem were frozen, while those containing atoms between 22 and 24 Å from N4 were subjected to a 25 kcal/mol harmonic restraint. The QM region consisted of up to 92 atoms including the entire biapenem, Asp120 (beyond CB), His118, His196, and His263 (beyond CB), Cys221 (beyond CA), Zn^2+^, and the water molecule hydrogen bonded to Asp120 and loosely coordinated to Zn^2+^. Hydrogen link atoms were placed at the boundaries between the QM and MM region. The QM region was treated by density functional theory (DFT) [45], [71] using the B3LYP functional [72] with the lacvp* basis set (with added “+” diffuse function only for the metal ion). In this basis set all atoms H through Ar are described with 6-31G*, while heavier atoms (e.g., Zn) are described using the LANL2DZ effective core potentials basis set. The MM region was represented with the 2005 OPLS-AA force-field.

Two-dimensional QM/MM potential energy surfaces (PESs) were constructed with relaxed scans (full geometry optimization at each scan point) employing either the C7_BIA_–O71_BIA/WAT1_ and C7_BIA_–N4_BIA_ bonds of the lactam ring (these are the C–O and C–N bonds that form and break, respectively, as the ring opens), or the C7_BIA_–O71_BIA/WAT1_ and N4_BIA_–HN4_ BIA_ bonds (this is the H–N bond that forms when N4^BIA^ is protonated) as coordinates for the scans. Points were obtained for each PES by constraining the grid coordinates (at 0.1 Å intervals) and minimizing the energy with respect to the remaining parameters. Minimum Energy Paths (MEP) on the PESs, providing the best possible approximation of the global reaction coordinate for the number of variables used in the scans, were calculated by a modification of the Zero Temperature String Method [73].

For the PES derived from **Simulation 3**, transition states (TSs) were refined by the quadratic synchronous transit method (QST) [74]–[76] utilizing two points of the PES on opposite sides of the TS, and confirmed to correspond to a single negative frequency. Total enthalpies (*H*) at 1 atm, 298.15 K for the TS(s), and all stationary points were calculated from the vibrational properties of the states as the sum of the total internal energy *U_tot_* (*U_tot_* = *QM/MM Energy*+*Zero Point Energy*+*U_trans_*+*U_rot_*+*U_vib_*) and the *pV* (pressure×volume) term. Total free energies (G) at 1 atm, 298.15 K were calculated also from the vibrational properties as *G* = *H* – T*S. A scaling factor of 0.9614 (as applicable to SCF calculations with B3LYP and 6-31G* [77]) and an inclusion threshold of 10.0 cm^−1^ were applied to vibrational frequencies prior to the calculation of thermochemical properties. Reaction and activation free energies *(ΔG^0^*, *ΔG* ^‡^) were calculated as the differences *G* ^PS^-*G* ^RS^, *G* ^TS^-*G* ^RS^ between the total free energies at the various stationary and transition states.

## Supporting Information

Figure S1 **PES and active site configurations for the reaction corresponding to simulation 1 in** **Table 2** **. Top left.** PES of the reaction calculated using the C7_BIA_–O71_BIA/WAT1_ and C7_BIA_–N4_BIA_ bonds as scanning coordinates. The minimum energy path (MEP) on the surface is traced by the beads-on-a-string white line. **Top right.** QM/MM energy values along the MEP. The other three insets show the configurations of the active site corresponding to the three numbered positions on the PES. At the RS (inset 1) the proton belongs to Wat1. Coincident with the formation of a tetrahedral intermediate, the proton is transferred from Wat1 to the hydroxide ion bound to Asp120, generating Wat2 (inset 2). Concurrent with the opening of the ring (inset 3) the proton is transferred from Wat2 to N4 of the β-lactam ring. Thus, the reaction proceeds through the formation of a tetrahedral intermediate, but the rate-limiting step is the protonation of the ring nitrogen (with a barrier of ∼20 kcal/mol). There is no proton transfer to His118, His196 or Asp120.
(TIF)

Figure S2 **PESs for the reactions corresponding to simulations 4–7 in** **Table 2** **. A.** PES of the reaction corresponding to **Simulation 4** in **Table 2**. **B.** PES of the reaction corresponding to **Simulation 5** in **Table 2**: two possible MEPs are shown. **C.** PES of the reaction corresponding to **Simulation 6** in **Table 2**. **D.** PES of the reaction corresponding to **Simulation 7** in **Table 2**.
(TIF)

Figure S3 **PES and active site configurations corresponding to simulation 8 in** **Table 2** **. Top left.** PES calculated using the C–O and H–N bonds as scanning coordinates. RS and PS are labeled 1 and 3, respectively. **Bottom left.** QM/MM energy values along the MEP. RS (labeled 1) and ionized PS (labeled 3) are separated by a barrier of ∼9 kcal/mol, and the reaction is strongly exergonic (−44 kcal/mol). The two insets on the right show the configurations of the active site at the RS and PS. Notice the unusual bicyclic compound representing the RS of the reaction (inset 1). The proximity of O3’ to C7 explains the intrinsic reactivity of this compound and its decay into a structure identical to hydrolyzed biapenem (inset 3).
(TIF)

Figure S4 **PESs and active site configurations for the reaction corresponding to simulation 10 in** **Table 2** **.** **Top left.** PES of the reaction calculated using the C–O and H–N bonds as scanning coordinates. Two equivalent MEPs are possible on this surface. **Top right.** QM/MM energy values along the two MEPs: the reaction is exergonic (−18 kcal/mol), and a barrier of ∼17 kcal/mol (labeled 2) separates RS (labeled 1) from PS (labeled 3). The other three insets show the configurations of the active site corresponding to the RS, TS, and PS states labeled 1,2,3 on the PES.
(TIF)

Figure S5 **PESs for the reaction corresponding to Simulations 11–13 in** **Table 2** **. A.** PES of the reaction corresponding to **simulation 11** in **Table 2**. **B.** PES of the reaction corresponding to **simulation 12** in **Table 2**: two possible MEPs are shown. **C.** PES of the reaction corresponding to **Simulation 13** in **Table 2**.
(TIF)

Figure S6 **Simulated steady-state kinetics of biapenem inactivation by CphA. Top left.** Calculated progress curves for the steady-state hydrolysis of 500 µM biapenem by 0.05 µM CphA, based on the kinetic model shown in **Figure 6**. Biapenem (sBIA, blue trace) is initially converted to its open-ring form with N4 protonated (s_open_NH4, red trace); however, very soon the open ring form is converted to the bicyclic derivative with N4 protonated (s_bicyc_NH4, purple trace, 95% of the total product) or with N4 deprotonated (s_bicyc_N4, cyan trace, 5% of the total product). The open-ring form of biapenem with N4 ionized (s_open_N4, green trace) is produced in negligible amounts. As a consequence, by the end of the reaction the bicyclic derivative with N4 protonated is the only species bound to the enzyme, consistent with the crystal structure of CphA incubated with biapenem [14], which shows the bicyclic derivative to be the only species bound in the active site. **Top right.** Calculated progress curves for the steady-state hydrolysis of different initial amounts of biapenem (sBIA) catalyzed by 0.05 µM CphA. Progress curves were computed using a deterministic model with Matlab Simbiology. In this approach, the ordinary differential equations (ODE) stiff solver ode15s (Matlab ODE Suite) was used to evaluate numerically the time course defined by the differential equations that describe the model (**Text S2**). **Bottom left.** Initial velocities of biapenem disappearance as determined from the progress curves in the previous panel. **Bottom right.** Lineweaver-Burk plot of the initial velocities *versus* the initial concentrations of biapenem with least-square fit of *k*_cat_ and *K*_m_.
(TIF)

Text S1 **Details of simulations 1, 4–8, 10–13, as listed in** **Table 2** **.**
(DOCX)

Text S2 **Species, parameters, reactions, and corresponding ordinary differential equations describing the kinetic model shown in** **Figure 6** **.**
(DOCX)

Model S1 **CphA kinetic model in Matlab SimBiology format.**
(TAR)

Model S2 **CphA kinetic model in SBML format.**
(XML)
