# Supplementary figures and images for: Biapenem Inactivation by B2 Metallo β-Lactamases: Energy Landscape of the Hydrolysis Reaction
Source: PLoS One. 2013 Jan 24;8(1):e55136. doi: 10.1371/journal.pone.0055136 (PMC3556986; doi:10.1371/journal.pone.0055136)

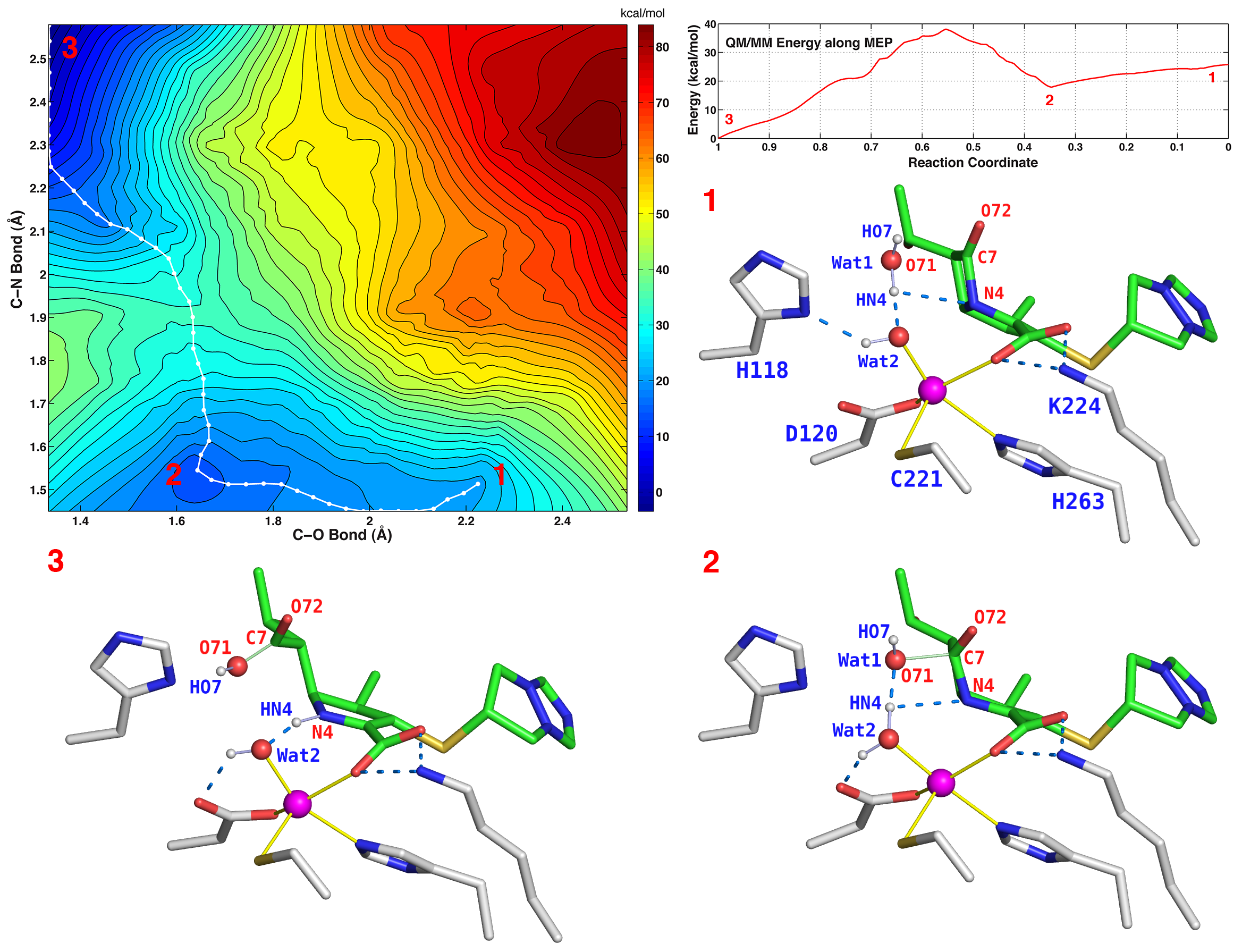

Supplement: Figure S1 — PES and active site configurations for the reaction corresponding to simulation 1 in Table 2 . Top left. PES of the reaction calculated using the C7BIA–O71BIA/WAT1 and C7BIA–N4BIA bonds as scanning coordinates. The minimum energy path (MEP) on the surface is traced by the beads-on-a-string white line. Top right. QM/MM energy values along the MEP. The other three insets show the configurations of the active site corresponding to the three numbered positions on the PES. At the RS (inset 1) the proton belongs to Wat1. Coincident with the formation of a tetrahedral intermediate, the proton is transferred from Wat1 to the hydroxide ion bound to Asp120, generating Wat2 (inset 2). Concurrent with the opening of the ring (inset 3) the proton is transferred from Wat2 to N4 of the β-lactam ring. Thus, the reaction proceeds through the formation of a tetrahedral intermediate, but the rate-limiting step is the protonation of the ring nitrogen (with a barrier of ∼20 kcal/mol). There is no proton transfer to His118, His196 or Asp120. (TIF) [file pone.0055136.s001.tif]

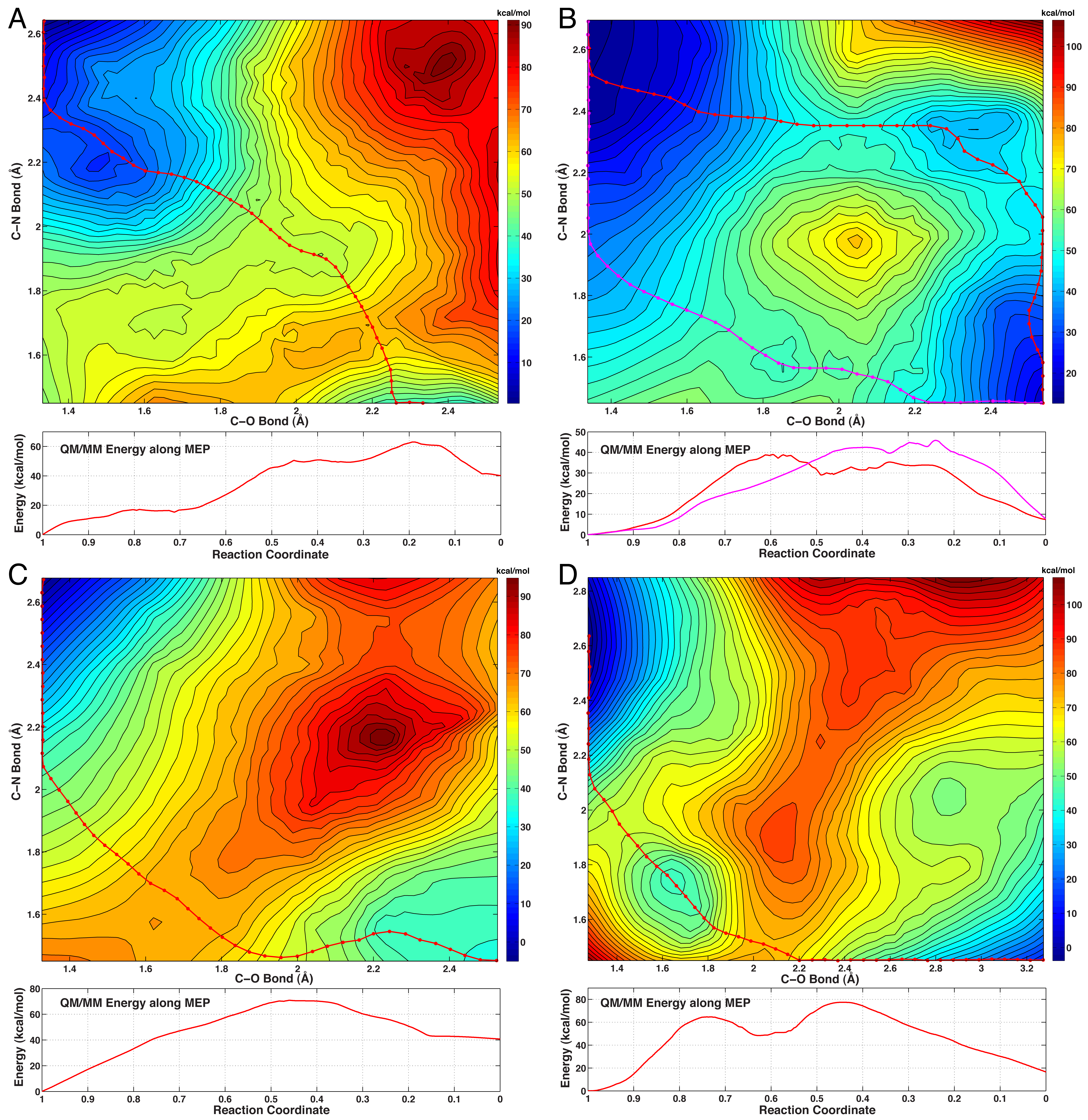

Supplement: Figure S2 — PESs for the reactions corresponding to simulations 4–7 in Table 2 . A. PES of the reaction corresponding to Simulation 4 in Table 2 . B. PES of the reaction corresponding to Simulation 5 in Table 2: two possible MEPs are shown. C. PES of the reaction corresponding to Simulation 6 in Table 2 . D. PES of the reaction corresponding to Simulation 7 in Table 2 . (TIF) [file pone.0055136.s002.tif]

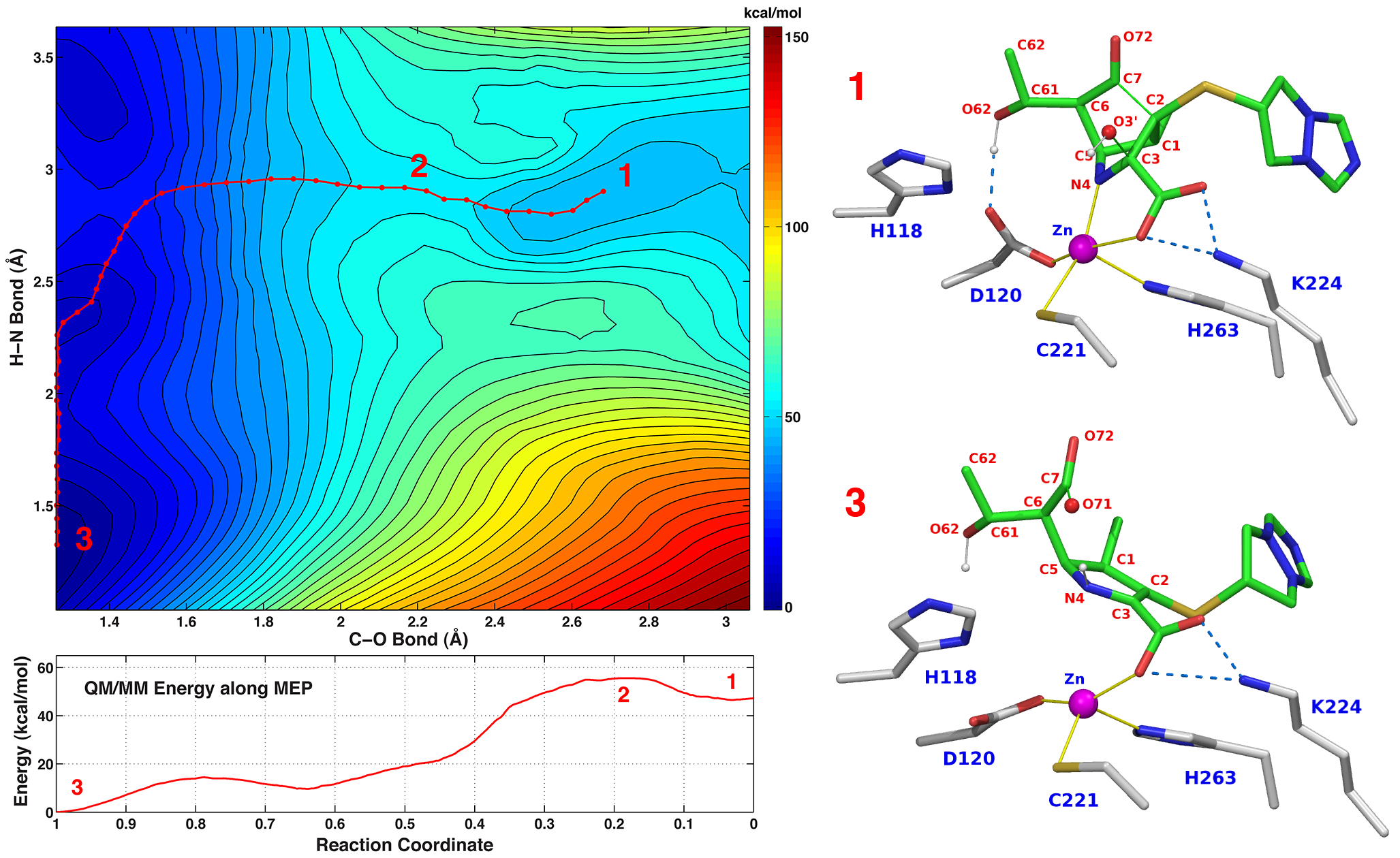

Supplement: Figure S3 — PES and active site configurations corresponding to simulation 8 in Table 2 . Top left. PES calculated using the C–O and H–N bonds as scanning coordinates. RS and PS are labeled 1 and 3, respectively. Bottom left. QM/MM energy values along the MEP. RS (labeled 1) and ionized PS (labeled 3) are separated by a barrier of ∼9 kcal/mol, and the reaction is strongly exergonic (−44 kcal/mol). The two insets on the right show the configurations of the active site at the RS and PS. Notice the unusual bicyclic compound representing the RS of the reaction (inset 1). The proximity of O3’ to C7 explains the intrinsic reactivity of this compound and its decay into a structure identical to hydrolyzed biapenem (inset 3). (TIF) [file pone.0055136.s003.tif]

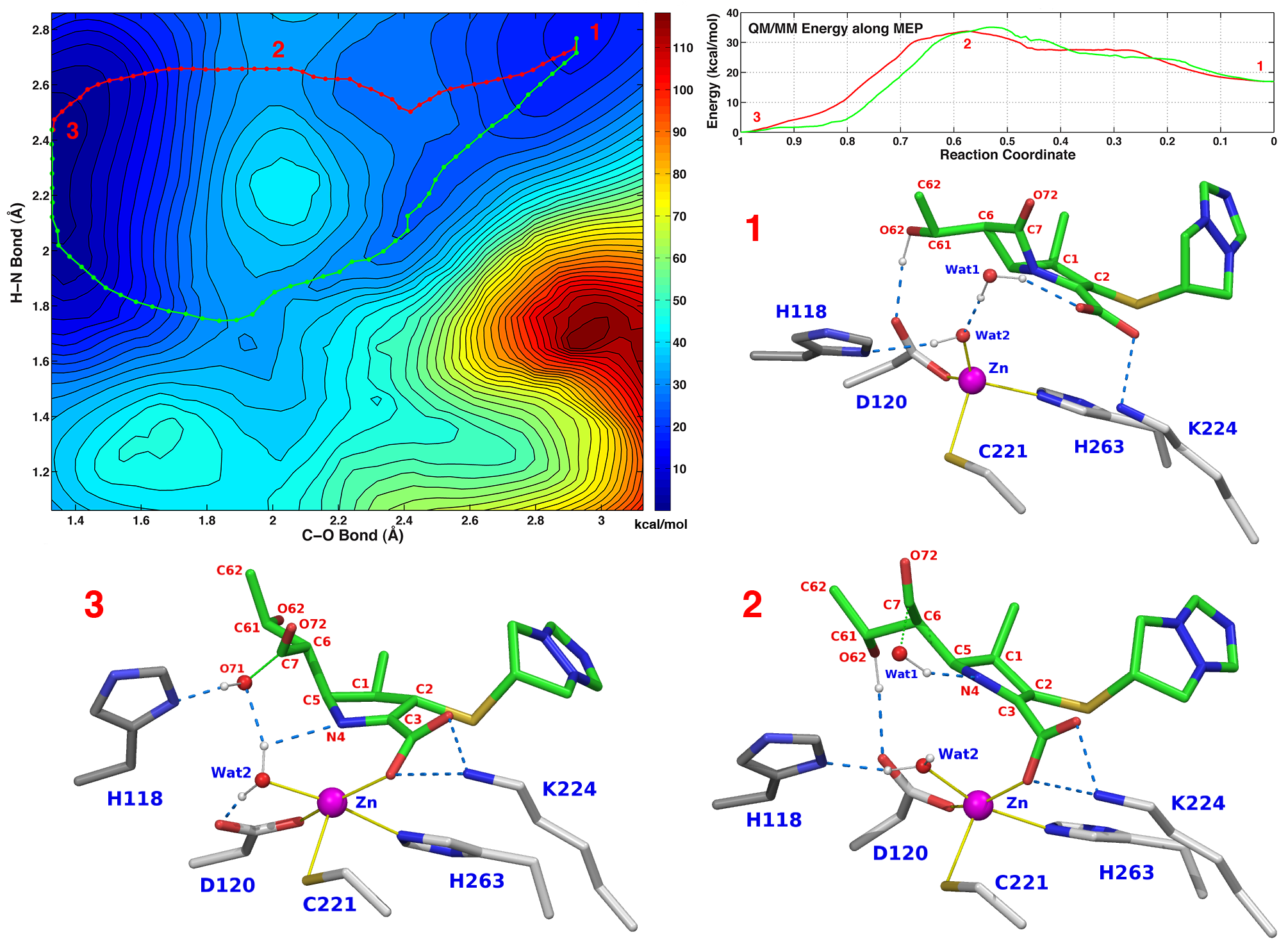

Supplement: Figure S4 — PESs and active site configurations for the reaction corresponding to simulation 10 in Table 2 . Top left. PES of the reaction calculated using the C–O and H–N bonds as scanning coordinates. Two equivalent MEPs are possible on this surface. Top right. QM/MM energy values along the two MEPs: the reaction is exergonic (−18 kcal/mol), and a barrier of ∼17 kcal/mol (labeled 2) separates RS (labeled 1) from PS (labeled 3). The other three insets show the configurations of the active site corresponding to the RS, TS, and PS states labeled 1,2,3 on the PES. (TIF) [file pone.0055136.s004.tif]

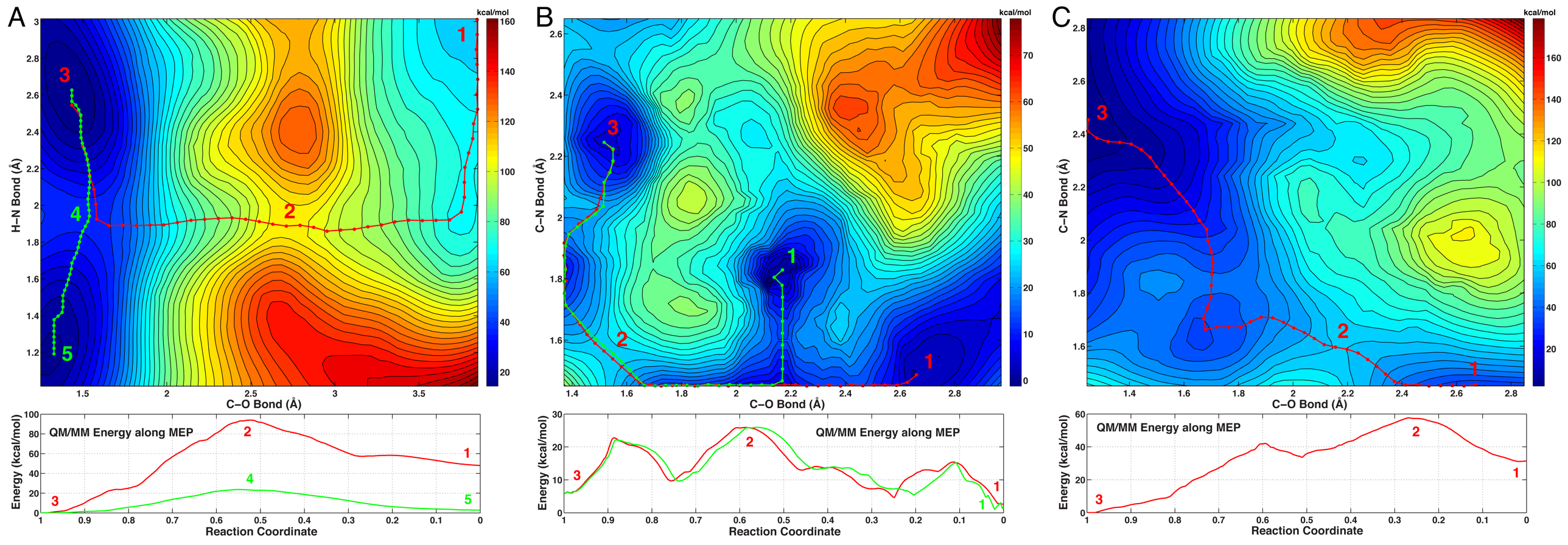

Supplement: Figure S5 — PESs for the reaction corresponding to Simulations 11–13 in Table 2 . A. PES of the reaction corresponding to simulation 11 in Table 2 . B. PES of the reaction corresponding to simulation 12 in Table 2: two possible MEPs are shown. C. PES of the reaction corresponding to Simulation 13 in Table 2 . (TIF) [file pone.0055136.s005.tif]

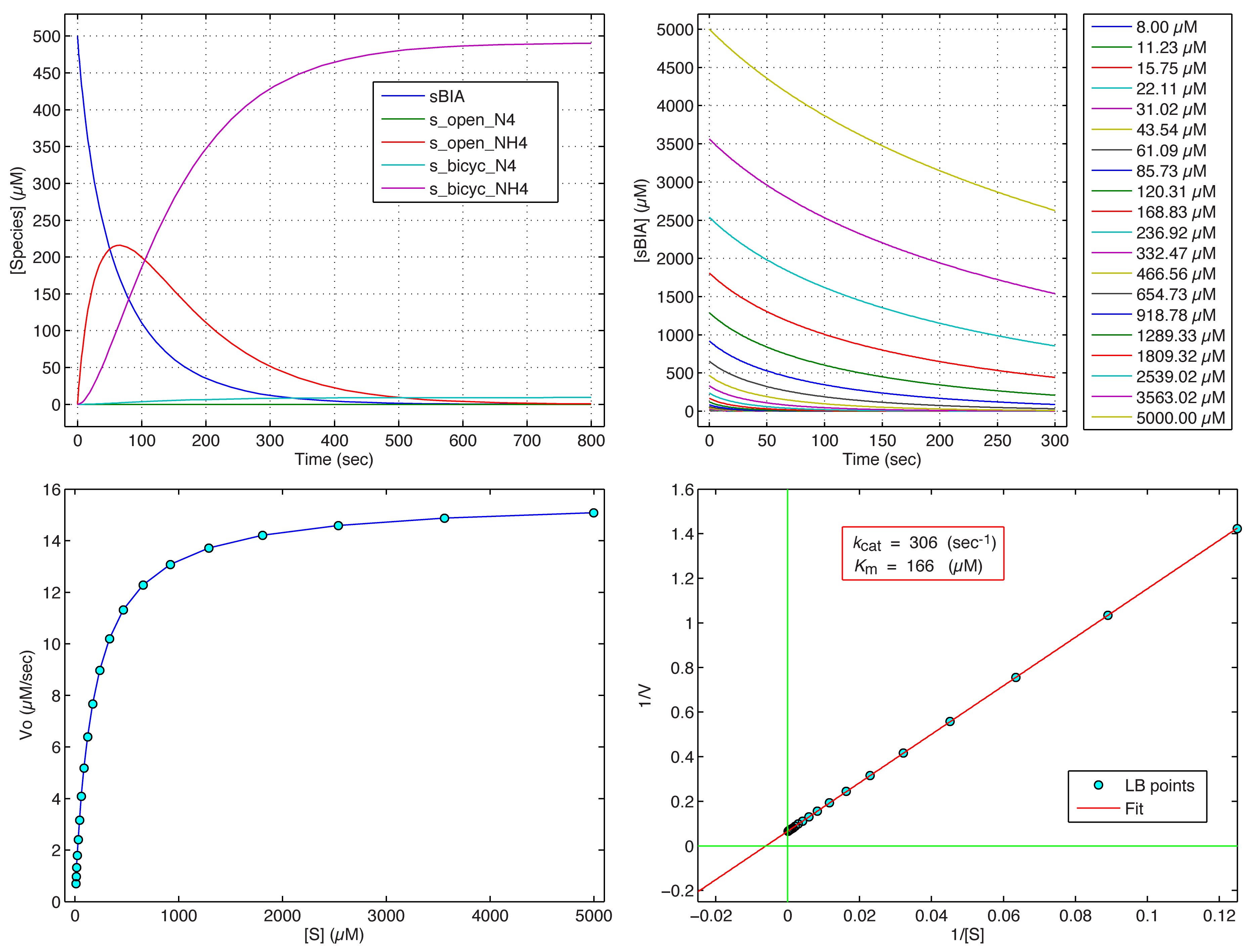

Supplement: Figure S6 — Simulated steady-state kinetics of biapenem inactivation by CphA. Top left. Calculated progress curves for the steady-state hydrolysis of 500 µM biapenem by 0.05 µM CphA, based on the kinetic model shown in Figure 6 . Biapenem (sBIA, blue trace) is initially converted to its open-ring form with N4 protonated (s_open_NH4, red trace); however, very soon the open ring form is converted to the bicyclic derivative with N4 protonated (s_bicyc_NH4, purple trace, 95% of the total product) or with N4 deprotonated (s_bicyc_N4, cyan trace, 5% of the total product). The open-ring form of biapenem with N4 ionized (s_open_N4, green trace) is produced in negligible amounts. As a consequence, by the end of the reaction the bicyclic derivative with N4 protonated is the only species bound to the enzyme, consistent with the crystal structure of CphA incubated with biapenem [14], which shows the bicyclic derivative to be the only species bound in the active site. Top right. Calculated progress curves for the steady-state hydrolysis of different initial amounts of biapenem (sBIA) catalyzed by 0.05 µM CphA. Progress curves were computed using a deterministic model with Matlab Simbiology. In this approach, the ordinary differential equations (ODE) stiff solver ode15s (Matlab ODE Suite) was used to evaluate numerically the time course defined by the differential equations that describe the model (Text S2). Bottom left. Initial velocities of biapenem disappearance as determined from the progress curves in the previous panel. Bottom right. Lineweaver-Burk plot of the initial velocities versus the initial concentrations of biapenem with least-square fit of k cat and K m. (TIF) [file pone.0055136.s006.tif]
