# Supplementary material for: Biapenem Inactivation by B2 Metallo β-Lactamases: Energy Landscape of the Hydrolysis Reaction
Source: PLoS One. 2013 Jan 24;8(1):e55136. doi: 10.1371/journal.pone.0055136 (PMC3556986; doi:10.1371/journal.pone.0055136)
Supplement: Text S1 — Details of simulations 1, 4–8, 10–13, as listed in Table 2 . (DOCX) [file pone.0055136.s007.docx]

**Text S1**

***Active site configuration 1.***

In a first experiment employing the C7_BIA_–O71_BIA/WAT1_ and C7_BIA_–N4_BIA_ bonds of the lactam ring as scanning coordinates (these are the C–O and C–N bonds that form and break, respectively, as the ring opens), we used the combination of ionization states at the product state PS in which Wat2 is deprotonated to a hydroxide ion, Asp120 is deprotonated, and His118, and His196 are neutral (**Table 2, Simulation 1**). **Figure S1** shows a very shallow downhill minimum energy path (MEP) from the reactant state RS (labeled **1** in the contour plot), in which Wat1 is far from the closed β-lactam ring (C–O≅2.2 Å, C–N≅1.5 Å), and a tetrahedral intermediate (labeled **2** in the contour plot) in which a hydroxide ion derived from Wat1 has attacked C7 of the β-lactam ring, but the ring is still closed (C–O≅1.65 Å, C–N≅1.5 Å). A barrier of ~20 kcal/mol separates the tetrahedral intermediate from the product state PS (labeled **3** in the contour plot), in which the ring is completely opened (C–O≅1.4 Å and C–N≅2.55 Å). A barrier of comparable height was calculated when the reaction was simulated using the C_BIA_–O_BIA/WAT1_, HN_BIA_–N_BIA_ distances as scanning coordinates in the construction of the PES (not shown).

***Active site configurations 4-5.***

Under different ionization states of the active site, the imidazole side chain of His118 may provide additional routes for protons to and from Wat1 and Wat2; His196 (see **Figure 1A**) can be a donor or acceptor of a hydrogen bond from Wat2 depending on whether the δ or ε nitrogen of the imidazole ring is protonated; and if Asp120 is protonated, Wat2 (or a hydroxide ion) will reorient to donate hydrogen bonds to both His118 and His196. Thus, for example, under conditions of the PS similar to those of **Simulation 1** of **Table 2**, but with either His196 protonated (**Table 2**, **Simulation 4**, **Figure S2A**), or with both His118 and His196 protonated (**Table 2**, **Simulation 5**, **Figure S2B**), barriers of ~24 and 30 kcal/mol, respectively, were calculated for the reaction from RS to PS.

***Active site configurations 6-8.***

The reaction was simulated under conditions consistent with the generally accepted mechanism of B2 MβLs, which predicts that only one water molecule (initially positioned near Asp120 at the Zn2 site) is the nucleophile that attacks the β-lactam ring. Since this water molecule is consumed in the reaction to form the product, testing this standard mechanism, requires that the Zn2 site be left empty at the PS when calculating the PES in a reverse scan from PS to RS.

In a first experiment of this type the C6 carboxylate and the N4 of biapenem were both protonated, and the Zn2 site was left empty in the PS configuration prior to carrying out the scan from product to substrate (**Table 2, Simulation 6**). As expected, at the RS (C–O≅2.6 Å, C–N≅1.5 Å, H–N≅3.06 Å) Wat2 is present (it is generated in the reverse reaction) and donates hydrogen bonds to both Asp120 and H118. At the TS (C–O≅1.7 Å, C–N≅1.6 Å, H–N≅1.03 Å) Wat2 splits with one proton going to biapenem N4, and the resulting hydroxide ion coordinating C7, which therefore displays a tetrahedral geometry. The calculated barrier for the reaction is ~30 kcal/mol (**Figure S2C**).

In a second experiment (**Table 2, Simulation 7**, **Figure S2D**), the Zn2 site was again left empty in the PS state, and N4^BIA^ was protonated, but the C6-carboxylate of biapenem was deprotonated. In this case, a tetrahedral intermediate (C–O≅1.64 Å, C–N≅1.74 Å, H–N≅2.5 Å) is formed on the minimum energy path (MEP) between RS (C–O≅3.3 Å, C–N≅1.45 Å, H–N≅3.7 Å) and PS (C–O≅1.27 Å, C–N≅2.6 Å, H–N≅1.04 Å), with a larger barrier of ~60 kcal/mol between RS and the intermediate and a smaller one of ~16 kcal/mol between the intermediate and PS (**Figure S2D**).

A third experiment was carried out by changing only the tautomeric state of His196 from HIE to HID (**Table 2, Simulation 8)**. This small change is sufficient to dramatically alter the course of the reverse reaction such that the derived RS is characterized by an unusual structure in which there is a hydroxyl group at C3, and a five-membered ring, comprising a bond between C7 and C2, replaces the standard β-lactam ring (**Figure S3**). While the barrier between RS and PS is only 9 kcal/mol, this PES clearly reflects the hydrolysis of a compound quite different from biapenem, although the product is the same.

***Active site configuration 10.***

A result similar to that obtained for Simulation 2 (in which Wat2 is protonated, biapenem N4 is deprotonated, and His196 is in the ε tautomeric state) was obtained when the simulation started from a PS (C–O≅1.34 Å, C–N≅2.6 Å) in which His196 is in the δ tautomeric state (Table 2, Simulation 10). In this case a barrier of ~17 kcal/mol was determined when the PES was calculated using the C–O, H–N distances as scanning coordinates (Figure S4). A barrier of similar height was derived when the PES was calculated using the C–O, C–N distances as scanning coordinates. Like in other simulations, a proton relay occurs between Wat1 and Wat2 as the reaction progresses: in the reactant state (C–O≅3.0 Å, C–N≅1.5 Å; labeled 1 in Figure S4) WAT1 is protonated and donates hydrogen bonds to Wat2 (present as OH^-^), biapenem C2 carboxylate, and to NE2 of HID196 (not shown). Wat2 is deprotonated with its only proton involved in a hydrogen bond with ND1 of His118. The TS of the reaction coincides with the formation of a tetrahedral species at C7 (C–O≅2.1 Å, C–N≅2.6 Å; labeled 2 in Figure S4), which occurs when Wat1 donates a proton to Wat2 and the resulting hydroxide ion attacks the carbonyl carbon of biapenem.

***Active site configuration 11.***

A PES calculated starting from a PS configuration very similar to configuration 10, but with a hydroxide ion instead of water at the Zn2 site (**Table 2**, **Simulation 11**), is shown in **Figure S5A**. While the scan started with a PS corresponding to deprotonated biapenem (C–O≅1.45 Å, H–N≅2.6 Å, labeled 3), the PES shows that the protonated form of biapenem (C–O≅1.4 Å, H–N≅1.2 Å, labeled 5) is equally probable as the reaction product. A barrier of ~46 kcal/mol separates the RS (C–O≅3.7 Å, C–N≅1.42 Å, H–N≅3.0 Å) from either form of PS.

***Active site configurations 12-13.***

In these PS configurations Asp120 was protonated and donated a hydrogen bond to Wat2. In the first configuration (**Table 2**, **Simulation 12**) the C6 carboxylate was protonated, N4 was deprotonated, and both His118 and His196 were in the HIE tautomeric state. At the PS configuration with the lowest energy, Wat2 donates hydrogen bonds to His118 and biapenem O71 and accepts hydrogen bonds from Asp120 and His196. The PES of the reaction (**Figure S5B**) is characterized by multiple approximately isoenergetic minima with a barrier of ~23 kcal/mol (at C–O≅1.53 Å, C–N≅1.56 Å) on the MEP connecting either one of two equivalent RSs to the PS (C–O≅1.53 Å, C–N≅2.25 Å). At the TS Wat1 is deprotonated and already part of a tetrahedral center at biapenem C7, while a fully protonated Wat2 donates hydrogen bonds to Wat1 and His118, and accepts a hydrogen bond from the protonated carboxylate moiety of Asp120. Notice that while in this simulation RS and PS are similar to those of **Simulation 3**, the scanning coordinates were the C–O and C–N distances instead of the C–O, H–N distances. This example highlights how the choice of scanning coordinates can be very important in identifying the lowest energy path between reactant and product.

In a second configuration (**Table 2**, **Simulation 13**) the C6 carboxylate was deprotonated at the PS (C–O≅1.3 Å, C–N≅2.45 Å), as expected to happen if a hydroxide ion attacks the carbonyl carbon of the β-lactam ring and its only proton is then transferred to the N4 atom. The calculated barrier on the PES starting from RS (at C–O≅2.7 Å, C–N≅1.5 Å) to this PS configuration was ~26 kcal/mol (**Figure S5C**).
