# Supplementary material for: Biapenem Inactivation by B2 Metallo β-Lactamases: Energy Landscape of the Hydrolysis Reaction
Source: PLoS One. 2013 Jan 24;8(1):e55136. doi: 10.1371/journal.pone.0055136 (PMC3556986; doi:10.1371/journal.pone.0055136)
Supplement: Text S2 — Species, parameters, reactions, and corresponding ordinary differential equations describing the kinetic model shown in Figure 6 . (DOCX) [file pone.0055136.s008.docx]

**Text S2.** Reactions, species, parameters, and corresponding ordinary differential equations describing the kinetic model shown in **Figure 6.**

Units

Species: μM

Rate Constants:

1^st^ order: s^-1^

2^nd^ order: μM^-1^s^-1^

Reactions

1. sBIA + CphA <-> e_BIA

biapenem binds to CphA

1. e_BIA <-> e_open_N4_INT

Wat1 attacks biapenem C7 and the β-lactam ring opens; N4 is ionized

1. e_open_N4_INT <-> e_open_N4

Wat1 approaches N4 of the open-ring form of biapenem

1. e_open_N4 <-> e_open_NH4
2. s_open_N4 <-> s_open_NH4

N4 of hydrolyzed biapenem is protonated

6 e_open_N4 <-> e_rot_N4

7 s_open_N4 <-> s_rot_N4

the hydroxyethyl group of hydrolyzed biapenem with N4 ionized rotates around the C5-C6 bond

8 e_open_NH4 <-> e_rot_NH4

9 s_open_NH4 <-> s_rot_NH4

the hydroxyethyl group of hydrolyzed biapenem with N4 protonated rotates around the C5-C6 bond

10 e_rot_N4 <-> e_protC2_N4

11 s_rot_N4 <-> s_protC2_N4

proton transfer from the hydroxyethyl group to C2

12 e_protC2_N4 <-> e_bicyclic_N4

13 s_protC2_N4 <-> s_bicyclic_N4

nucleophilic attack on C3 by the oxygen atom of the hydroxyethyl group leading to the formation of a bicyclic compound with N4 ionized

14 e_bicyclic_N4 <-> e_bicyclic_BIA_NH4

15 s_bicyclic_N4 <-> s_bicyclic_BIA_NH4

the N4 of the bicyclic compound is protonated

16 e_rot_N4 <-> e_rot_NH4

17 s_rot_N4 <-> s_rot_NH4

protonation of biapenem N4 after the rotation of the hydroethyl group has already occurred

18 e_rot_NH4 <-> e_bicyclic_BIA_NH4

19 s_rot_NH4 <-> s_bicyclic_BIA_NH4

direct cyclization of hydrolyzed biapenem with N4 protonated and with the hydroxyethyl group rotated

20 e_open_N4 <-> s_open_N4 + CphA

hydrolyzed biapenem with N4 ionized leaves the enzyme

21 e_open_NH4 <-> s_open_NH4 + CphA

hydrolyzed biapenem with N4 protonated leaves the enzyme

22 e_rot_N4 <-> s_rot_N4 + CphA

hydrolyzed biapenem with N4 ionized and with the hydroxyethyl group rotated leaves the enzyme

23 e_rot_NH4 <-> s_rot_NH4 + CphA

hydrolyzed biapenem with N4 protonated and with the hydroxyethyl group rotated leaves the enzyme

24 e_protC2_N4 <-> s_protC2_N4 + CphA

hydrolyzed biapenem with N4 ionized and with the rotated hydroxyethyl group that has already transferred a proton to C2 leaves the enzyme

25 CphA + s_bicyclic_BIA_NH4 <-> e_bicyclic_BIA_NH4

the bicyclic form of biapenem with N4 protonated binds to the enzyme

26 e_bicyclic_N4 <-> s_bicyclic_N4 + CphA

the bicyclic form of biapenem with N4 ionized leaves the enzyme

ODEs

d(e_BIA)/dt = 1/Solution*(ReactionFlux1 - ReactionFlux2)

d(e_open_N4_INT)/dt = 1/Solution*(ReactionFlux2 - ReactionFlux3)

d(e_open_N4)/dt = 1/Solution*(ReactionFlux3 - ReactionFlux4 - ReactionFlux6 - ReactionFlux20)

d(e_open_NH4)/dt = 1/Solution*(ReactionFlux4 - ReactionFlux8 - ReactionFlux21)

d(e_rot_N4)/dt = 1/Solution*(ReactionFlux6 - ReactionFlux10 - ReactionFlux16 - ReactionFlux22)

d(e_protC2_N4)/dt = 1/Solution*(ReactionFlux10 - ReactionFlux12 - ReactionFlux24)

d(e_bicyclic_N4)/dt = 1/Solution*(ReactionFlux12 - ReactionFlux14 - ReactionFlux26)

d(e_bicyclic_BIA_NH4)/dt = 1/Solution*(ReactionFlux14 + ReactionFlux18 + ReactionFlux25)

d(e_rot_NH4)/dt = 1/Solution*(ReactionFlux8 + ReactionFlux16 - ReactionFlux18 - ReactionFlux23)

d(s_open_N4)/dt = 1/Solution*(-ReactionFlux5 - ReactionFlux7 + ReactionFlux20)

d(s_rot_N4)/dt = 1/Solution*(ReactionFlux7 - ReactionFlux11 - ReactionFlux17 + ReactionFlux22)

d(s_open_NH4)/dt = 1/Solution*(ReactionFlux5 - ReactionFlux9 + ReactionFlux21)

d(s_rot_NH4)/dt = 1/Solution*(ReactionFlux9 + ReactionFlux17 - ReactionFlux19 + ReactionFlux23)

d(s_bicyclic_BIA_NH4)/dt = 1/Solution*(ReactionFlux15 + ReactionFlux19 - ReactionFlux25)

d(s_bicyclic_N4)/dt = 1/Solution*(ReactionFlux13 - ReactionFlux15 + ReactionFlux26)

d(s_protC2_N4)/dt = 1/Solution*(ReactionFlux11 - ReactionFlux13 + ReactionFlux24)

d(sBIA)/dt = 1/Solution*(-ReactionFlux1)

d(CphA)/dt = 1/Solution*(-ReactionFlux1 + ReactionFlux20 + ReactionFlux21 + ReactionFlux22 + ReactionFlux23 + ReactionFlux24 - ReactionFlux25 + ReactionFlux26)

Fluxes

ReactionFlux1 = (kbind_1*sBIA*CphA)*Solution-(kbind_m1*e_BIA)*Solution

ReactionFlux2 = (k1*e_BIA)*Solution-(km1*e_open_N4_INT)*Solution

ReactionFlux3 = (k2*e_open_N4_INT)*Solution-(km2*e_open_N4)*Solution

ReactionFlux4 = (k3*e_open_N4)*Solution-(km3*e_open_NH4)*Solution

ReactionFlux5 = (kprot_s_open1*s_open_N4)*Solution-(kprot_s_openm1*s_open_NH4)*Solution

ReactionFlux6 = (krot_e1*e_open_N4)*Solution-(krot_em1*e_rot_N4)*Solution

ReactionFlux7 = (krot1*s_open_N4)*Solution-(krotm1*s_rot_N4)*Solution

ReactionFlux8 = (krot_e2*e_open_NH4)*Solution-(krot_em2*e_rot_NH4)*Solution

ReactionFlux9 = (krot2_1*s_open_NH4)*Solution-(krot2_m1*s_rot_NH4)*Solution

ReactionFlux10 = (e_C2_protonation.kprot_e1*e_rot_N4)*Solution-(e_C2_protonation.kprot_e2*e_protC2_N4)*Solution

ReactionFlux11 = (bk1*s_rot_N4)*Solution-(bkm1*s_protC2_N4)*Solution

ReactionFlux12 = (k_C3_e1*e_protC2_N4)*Solution-(k_C3_em1*e_bicyclic_N4)*Solution

ReactionFlux13 = (bk2*s_protC2_N4)*Solution-(bkm2*s_bicyclic_N4)*Solution

ReactionFlux14 = (e_bicyclic_N4_protonation.kprot_e2*e_bicyclic_N4)*Solution-(kprot_em2*e_bicyclic_BIA_NH4)*Solution

ReactionFlux15 = (bk3*s_bicyclic_N4)*Solution-(bkm3*s_bicyclic_BIA_NH4)*Solution

ReactionFlux16 = (e_rot_N4_protonation.kprot_e1*e_rot_N4)*Solution-(kprot_em1*e_rot_NH4)*Solution

ReactionFlux17 = (kprot1*s_rot_N4)*Solution-(kprotm1*s_rot_NH4)*Solution

ReactionFlux18 = (k_ecyc1*e_rot_NH4)*Solution-(k_ecycm1*e_bicyclic_BIA_NH4)*Solution

ReactionFlux19 = (kcyc*s_rot_NH4)*Solution-(kcycm1*s_bicyclic_BIA_NH4)*Solution

ReactionFlux20 = (kout*e_open_N4)*Solution-(kin*s_open_N4*CphA)*Solution

ReactionFlux21 = (kout_2*e_open_NH4)*Solution-(kin_2*s_open_NH4*CphA)*Solution

ReactionFlux22 = (kout_rotopenN4*e_rot_N4)*Solution-(kin_rotopenN4*s_rot_N4*CphA)*Solution

ReactionFlux23 = (kout_rotopenNH4*e_rot_NH4)*Solution-(kin_rotopenNH4*s_rot_NH4*CphA)*Solution

ReactionFlux24 = (kout_eprotC2*e_protC2_N4)*Solution-(kin_eprotC2*s_protC2_N4*CphA)*Solution

ReactionFlux25 = (kbind_b1*CphA*s_bicyclic_BIA_NH4)*Solution-(kbind_bm1*e_bicyclic_BIA_NH4)*Solution

ReactionFlux26 = (kout_bicyc_N4*e_bicyclic_N4)*Solution-(kin_bicyc_N4*s_bicyclic_N4*CphA)*Solution

Parameter Values

kbind_1 = 10

kbind_m1 = 2500

k1 = 599.18

km1 = 5.6966e-16

k2 = 1.6736e+10

km2 = 7.1177e-06

k3 = 5.2495e-09

km3 = 1.7322

kprot_s_open1 = 1e+10

kprot_s_openm1 = 1

krot_e1 = 9809.1

krot_em1 = 201.67

krot1 = 1.6832e+10

krotm1 = 6.0092e+06

krot_e2 = 16.012

krot_em2 = 2.9228e+08

krot2_1 = 52.22

krot2_m1 = 1.3982e+07

e_C2_protonation.kprot_e1 = 4.2648e+05

e_C2_protonation.kprot_e2 = 3.8933e+05

bk1 = 1.6893e+10

bkm1 = 5.712e+11

k_C3_e1 = 1.0147e+09

k_C3_em1 = 191.84

bk2 = 70683

bkm2 = 1.6244e-05

e_bicyclic_N4_protonation.kprot_e2 = 6.178e+11

kprot_em2 = 1.0898e-11

bk3 = 2.2037e+12

bkm3 = 4.1507e+10

e_rot_N4_protonation.kprot_e1 = 6.1341e+12

kprot_em1 = 5.9087e-07

kprot1 = 6.0466e+10

kprotm1 = 1.0337e+11

k_ecyc1 = 0.00022641

k_ecycm1 = 2.1716e-23

kcyc = 0.0023

kcycm1 = 5.2412e-11

kout = 650

kin = 10

kout_2 = 650

kin_2 = 10

kout_rotopenN4 = 10000

kin_rotopenN4 = 10

kout_rotopenNH4 = 600

kin_rotopenNH4 = 10

kout_eprotC2 = 6000

kin_eprotC2 = 10

kbind_b1 = 10

kbind_bm1 = 600

kout_bicyc_N4 = 10000

kin_bicyc_N4 = 10

Initial Conditions

e_BIA = 0

e_open_N4_INT = 0

e_open_N4 = 0

e_open_NH4 = 0

e_rot_N4 = 0

e_protC2_N4 = 0

e_bicyclic_N4 = 0

e_bicyclic_BIA_NH4 = 0

e_rot_NH4 = 0

s_open_N4 = 0

s_rot_N4 = 0

s_open_NH4 = 0

s_rot_NH4 = 0

s_bicyclic_BIA_NH4 = 0

s_bicyclic_N4 = 0

s_protC2_N4 = 0

sBIA = 500

CphA = 0.05
